# Supplementary material for: Effectiveness and cost-effectiveness of a sustainable obesity prevention programme for preschool children delivered at scale ‘HENRY’ (Health, Exercise, Nutrition for the Really Young): protocol for the HENRY III cluster randomised controlled trial
Source: BMJ Open. 2024 Mar 25;14(3):e081861. doi: 10.1136/bmjopen-2023-081861 (PMC10966824; doi:10.1136/bmjopen-2023-081861)
Supplement: Supplementary data [file bmjopen-2023-081861supp001.pdf]

Supplementary Table 1 Data collection summary

**Data/outcomes**	**Measures**	Collected by	LA[c] / CC[d] Screening and consent	Centre baseline	Participant screening	Participant informed consent and registration	Participant / staff Baseline	Short term follow-up (12 months post registration)	Medium-term follow-up (3 years)[a]	Longer term follow-up[b]
**Local authority**										
Eligibility	LA Eligibility checklist	LA self complete	x							
Basic demographics / reasons for declining	LA / CC Recruitment log	Researcher / HENRY	x							
Centre contact details	Centre nomination form	LA self complete	x							
Local authority agreement / consent	Local authority agreement	LA self complete	x							
**Centre**										
Eligibility	LA Eligibility checklist	LA self complete	x							
Reasons for declining / ineligibility	LA / CC recruitment log	Researcher	x							
Centre agreement / consent	Children’s Centre agreement	Centre self complete	x							

**Data/outcomes**	**Measures**	Collected by	LA[c] / CC[d] Screening and consent	Centre baseline	Participant screening	Participant informed consent and registration	Participant / staff Baseline	Short term follow-up (12 months post registration)	Medium-term follow-up (3 years)[a]	Longer term follow-up[b]
Stratification factors	Children's centre nomination form	LA self complete		x						
Centre randomisation allocation	Randomisation form	CTRU[e]		x						
Demographics	Centre nomination form / Environmental Questionnaire	LA / Centre self complete		x						
Details of other courses	Environmental Questionnaire	Centre self complete		x				x	x	
**Parent**										
Initial eligibility check	Screening form	Centre staff			x					
Confirmation of eligibility	Eligibility checklist	LCRN[f]				x				
Reasons for declining / ineligibility	Screening form	Parent self complete			x					
Demographics (parent, child and family)	Screening form<br>Baseline form	Parent self complete<br>CTRU (recruitment log)			x		x			

**Data/outcomes**	**Measures**	Collected by	LA[c] / CC[d] Screening and consent	Centre baseline	Participant screening	Participant informed consent and registration	Participant / staff Baseline	Short term follow-up (12 months post registration)	Medium-term follow-up (3 years)[a]	Longer term follow-up[b]
Consent to trial participation and data collection (including NCMP)	Consent form	Parent self complete				x				
Use of Centres	Baseline form<br>Follow up form	LCRN					x	x	x	
**Outcomes**										
Child age and sex adjusted BMI Z-score, child height, child weight, unadjusted BMI and weight/BMI percentiles	Measured	LCRN (baseline & 12 M follow-up)<br><br>Longer term: CTRU accessing routine data					x	x	x	
Sibling age and sex adjusted BMI, height (m), weight (kg), unadjusted BMI and weight/BMI percentiles		CTRU accessing routine data							x	
Parent self-efficacy	Dumka (27)	LCRN					x	x		
Family eating / activities	Golan (28)	LCRN					x	x		
Feeding questionnaire	Baughcum (29)	LCRN					x	x		

**Data/outcomes**	**Measures**	Collected by	LA[c] / CC[d] Screening and consent	Centre baseline	Participant screening	Participant informed consent and registration	Participant / staff Baseline	Short term follow-up (12 months post registration)	Medium-term follow-up (3 years)[a]	Longer term follow-up[b]
Dental health (child)	Dental questionnaire	LCRN					x	x		
Parent height & weight:	Measured	LCRN					x	x		
Parent waist circumference	Measured	LCRN					x	x		
**Staff:**										
Staff screening	Staff screening form	Staff self complete at centre		x						
Staff height & weight	Measured	Self-measure					x	x		
Staff waist circumference	Measured	Self-measure					X	x		
**Children's Centre:**										
Centre social, physical and political environment (e.g. policies around food)	Environmental questionnaire	Centre self complete		x				x		
**Safety:**										

**Data/outcomes**	**Measures**	Collected by	LA[c] / CC[d] Screening and consent	Centre baseline	Participant screening	Participant informed consent and registration	Participant / staff Baseline	Short term follow-up (12 months post registration)	Medium-term follow-up (3 years)[a]	Longer term follow-up[b]
Adverse events / unintended consequences	SAE / RUSAE forms	LCRN / centre self complete						x		
**Health economics:**										
Health care resource use for child	Resource use questionnaire	LCRN					x	x		
Health care resource use for parents	Resource use questionnaire	LCRN					x	x		
Private costs (e.g. travel costs, additional food expenses, lost productivity)	Resource use questionnaire	LCRN					x	x		
Parent's Health-related Quality of Life	EQ-5D-5L (30) and ICECAP-A [31]	LCRN					x	x		
**Routine data:**										
NCMP Child and sibling data (trial participants)		CTRU							x	
NCMP regional child data (not trial participants)		CTRU							x	

BMJ Publishing Group Limited (BMJ) disclaims all liability and responsibility arising from any reliance placed on this supplemental material which has been supplied by the author(s)

**Data/outcomes**	**Measures**	Collected by	LA[c] / CC[d] Screening and consent	Centre baseline	Participant screening	Participant informed consent and registration	Participant / staff Baseline	Short term follow-up (12 months post registration)	Medium-term follow-up (3 years)[a]	Longer term follow-up[b]
Process evaluation/delivery of HENRY programmes:										
Attendance * *Data collection not timed to trial time points	HENRY attendance records	HENRY Centre staff / HENRY facilitator								
Contamination checks* *Data collection not timed to trial time points	Staff movement form	Staff self complete								
HENRY training attendance and knowledge		HENRY						x	x	
**Qualitative data**										
System map and sub maps* *Data collection not timed to trial time points	n/a	Researcher								
Systems mapping interviews with local authority stakeholders* *Data collection not timed to trial time points	n/a	Researcher								

**Data/outcomes**	**Measures**	Collected by	LA[c] / CC[d] Screening and consent	Centre baseline	Participant screening	Participant informed consent and registration	Participant / staff Baseline	Short term follow-up (12 months post registration)	Medium-term follow-up (3 years)[a]	Longer term follow-up[b]
Interviews with centre staff* *Data collection not timed to trial time points	n/a	Researcher								
Interviews with parents* **Data collection not timed to trial time points	n/a	Researcher								

[a] Medium term outcomes will gathered from routinely collected data (from health visitors and/or the National Child Measurement Programme)

[b] Longer term outcomes will be based on matched cohorts of (Millennium Cohort Study (MCS ((21)).

[C] Local Authority (or other governing/commissioning body)

[d] Children's centre (or other setting in which HENRY can be delivered, including Sure Start centres in Northern Ireland)

[e] Clinical Trials Research Unit

[f] Local Clinical Research Network (England only). Other devolved nations will train researchers/equivalent staff to collect data)

BMJ Publishing Group Limited (BMJ) disclaims all liability and responsibility arising from any reliance placed on this supplemental material which has been supplied by the author(s)
