# Supplementary material for: Effectiveness and cost-effectiveness of a sustainable obesity prevention programme for preschool children delivered at scale ‘HENRY’ (Health, Exercise, Nutrition for the Really Young): protocol for the HENRY III cluster randomised controlled trial
Source: BMJ Open. 2024 Mar 25;14(3):e081861. doi: 10.1136/bmjopen-2023-081861 (PMC10966824; doi:10.1136/bmjopen-2023-081861)
Supplement: Author's manuscript [file bmjopen-2023-081861.draft_revisions.pdf]

BMJ Open is committed to open peer review. As part of this commitment we make the peer review history of every article we publish publicly available.

When an article is published we post the peer reviewers' comments and the authors' responses online. We also post the versions of the paper that were used during peer review. These are the versions that the peer review comments apply to.

The versions of the paper that follow are the versions that were submitted during the peer review process. They are not the versions of record or the final published versions. They should not be cited or distributed as the published version of this manuscript.

BMJ Open is an open access journal and the full, final, typeset and author-corrected version of record of the manuscript is available on our site with no access controls, subscription charges or pay-per-view fees (http://bmjopen.bmj.com).

If you have any questions on BMJ Open's open peer review process please email info.bmjopen@bmj.com

BMJ Open

Evaluation of a sustainable obesity prevention programme for pre-school children delivered at scale 'HENRY' (Health, Exercise, Nutrition for the Really Young): Protocol for the HENRY III Randomised Controlled Trial

Journal:	*BMJ Open*
Manuscript ID	bmjopen-2023-081861
Article Type:	Protocol
Date Submitted by the Author:	09-Nov-2023

Complete List of Authors:	Bryant, Maria; University of York, Hull York Medical School; University of York, Health Sciences<br>Burton, Wendy; University of York, Department of Health Sciences<br>Collinson, Michelle; University of Leeds Clinical Trials Research Unit<br>Martin, Adam; Academic Unit of Health Economics, Leeds Institute of Health Sciences<br>Copsey, Bethan; University of Leeds Clinical Trials Research Unit,<br>Groves-Williams, Dawn; University of Leeds Clinical Trials Research Unit,<br>Foster, Alexis; University of Sheffield, School of Health and Related Research<br>Willis, Thomas A; University of Leeds<br>Sharif, Amir; University of Bradford<br>O'Cathain, Alicia; University of Sheffield, School of Health and Related Research

Keywords:	Obesity, Community child health < PAEDIATRICS, Primary Prevention, PUBLIC HEALTH, Randomized Controlled Trial

SCHOLARONE™
Manuscripts

Evaluation of a sustainable obesity prevention programme for pre-school children delivered at scale 'HENRY' (Health, Exercise, Nutrition for the Really Young): Protocol for the HENRY III Randomised Controlled Trial

Professor Maria Bryant[1], Dr Wendy Burton[1], Ms Michelle Collinson[3], Dr Adam Martin[2], Dr Bethan Copsey[3], Dr Dawn Groves-Williams[3], Dr Alexis Foster[4]; Dr Thomas A Willis[3], Professor Amir Sharif[5], Professor Alicia O'Cathain[4]

[1] University of York, York, YO105DD

[2] Academic Unit of Health Economics, University of Leeds, LS29JT

[3] Clinical Trials Research Unit, Leeds Institute for Clinical Trials Research, University of Leeds, LS29JT

[4] Sheffield centre for Health and Related Research (ScHaRR), University of Sheffield, S1 4DA

[5] University of Bradford, BD7 1DP

Corresponding author: Professor Maria Bryant, maria.bryant@york.ac.uk

Collaborator: Kim Roberts, HENRY, 6 Elm Place, Old Witney Road, Oxfordshire, OX29 4BD

Keywords: Obesity, prevention, protocol, community, childhood, systems

Word count: 5436

For peer review only - http://bmjopen.bmj.com/site/about/guidelines.xhtml

Abstract

Introduction

A fifth of children start school with already living with overweight or obesity, with rates disproportionately impacting those living in the highest levels of deprivation. Social, environmental and biological factors contribute to excess weight gain and programmes delivered in early years settings aim to support families to navigate these in order to prevent obesity. One of these (HENRY) has been delivered in UK community venues (hereon named 'centres') in high deprivation areas since 2008 and aims to help families to provide a healthy start for their pre-school children. We aim to establish the effectiveness and cost-effectiveness of HENRY, including its potential role from a wider systems perspective.

Methods and analysis

This is a multi-centre, open-labelled, two group, prospective, cluster RCT, with cost effectiveness analysis, systems-based process evaluation and internal pilot. Primary analysis will compare BMI z-score at 12 months in children (n=984) whose parents have attended HENRY to those who have not attended. Secondary outcomes include parent and staff BMI and waist circumference, parenting-efficacy, feeding, eating habits, quality of life, resource use and medium term (3 year) BMI z-scores (child and siblings). Eighty-two centres in ~14 local authority areas will be randomised (1:1) to receive HENRY or continue with standard practice. Intention-to-treat analysis will compare outcomes using mixed effects linear regression. Economic evaluation will estimate a within-trial calculation of cost-per unit change in BMI z-score and longer-term trajectories to determine lifelong cost savings (long term outcomes). A systems process evaluation will explore whether (and how) implementation of HENRY impacts (and is impacted by) the early years obesity system. An established parent advisory group will support delivery and dissemination.

Ethics and dissemination

Ethical approval has been granted by the University of York, Health Sciences' Research Governance Committee (HSRGC/2022/537/E). Dissemination includes policy reports, community resources, social media and academic outputs.

Registration details

ISRCTN16529380

Strengths and limitations of this study

- The HENRY III trial provides an opportunity to examine the effectiveness and cost-effectiveness of an intervention that has been publicly funded and delivered at scale for more than 10 years, including short, medium and long-term outcomes
- Our systems-based process evaluation will explore the role that community-based obesity prevention interventions play within the wider system, based on the understanding that factors that influence obesity are complex and multifaceted.
- The HENRY III trial has been designed following a successful feasibility study, in collaboration with local and national government partners, members of the public and a multi-disciplinary trial team.

For peer review only - http://bmjopen.bmj.com/site/about/guidelines.xhtml

For peer review only

- The trial's success is dependent upon recruitment of local authority areas, where additional funding will be required to commission the intervention.
- Given the nature of the intervention, a cluster design was chosen to prevent contamination. Intervention allocation at the centre level means that there is a risk of selection bias, where parents/carers who are recruited from HENRY intervention centres may be characteristically different to those recruited from the control centres. A mitigation strategy has been developed to prevent this.

For peer review only

For peer review only - http://bmjopen.bmj.com/site/about/guidelines.xhtml

Introduction

Reducing the prevalence of childhood obesity amongst people living in deprived areas is a public policy priority. Since the COVID-19 pandemic, approximately one third of children (27.7% on average and up to 34.5% in higher deprivation areas) are defined as overweight (13.3%) or having obesity (14.4%) compared to 23.2% pre-COVID (1). This is the largest annual increase in the UK since the National Child Measurement Programme (NCMP) recording began. Health inequalities have also broadened, from a 6.3% difference in rates of overweight and obesity between the most and least deprived areas in 2019/20 to 10.7% in 2021/22 (1, 2).

Preventing excess weight in childhood is beneficial for health and wellbeing, as well as reducing medium- to long-term burden upon health services for those living with obesity (including mental health services and those used to treat and manage respiratory diseases such as asthma (3, 4). Obesity prevention during childhood can also reduce excess weight gain and obesity in later life (5, 6) which is difficult to reverse once established (7-9) and is a cause of numerous long-term, chronic health conditions. However, interventions to prevent obesity generally result in modest but inconsistent benefits (7-10). Given that obesity is caused by a wide range of factors, this is unsurprising and means that the role and cost-effectiveness of locally delivered programmes within a large and complex socio-economic and public health system is uncertain. Public Health England (PHE) advocates systems approaches; encouraging local areas to adopt a range of interconnecting interventions and policies inside and outside the healthcare sector to collectively tackle obesity and related health inequalities. It remains unknown what role individual interventions play in disrupting the system (11), and which of these interventions are most effective and cost-effective. Locally-delivered public health prevention programmes can be cost-effective (12); however, there is limited evidence specifically looking at obesity prevention delivered at scale.

One obesity prevention programme which has been delivered at scale for many years in the UK is HENRY (Health, Exercise and Nutrition for the Really Young); a community-based programme, designed to alter early years settings, upskill the early years workforce and improve lifestyle behaviours of parents/carers (hereon called ‘parents’) and their preschool aged (under 5 years old) children. Although HENRY was designed to be a universal programme, it has been predominantly delivered in children’s centres/community venues located in areas of high deprivation. Evidence suggests it has potential to impact on population obesity (13) but its effectiveness is not yet established. The current trial adopts a novel approach to evaluate the effectiveness and cost-effectiveness of the programme and explore the potential role of HENRY in disrupting the multiple factors that influence excess weight gain in the system. This novel approach places an emphasis on exploring if and how HENRY influences the system in which it operates (including system balancers which may reduce its potential for population impact).

Following on from a successful feasibility study (13), our current evaluation will consider child and sibling obesity outcomes in the short-term (using data collected in the trial), medium-term (using routinely collected data at 3 years) and longer-term (using economic modelling and secondary datasets).

Aims and objectives

Aim

To establish the effectiveness and cost-effectiveness of an obesity prevention programme delivered at scale, including its potential role from a wider systems perspective.

For peer review only - http://bmjopen.bmj.com/site/about/guidelines.xhtml

Primary objective of Randomised Controlled Trial (RCT)

1. The primary objective of the trial is to determine whether HENRY reduces child age- and sex - adjusted Body Mass Index (BMI) at 12 months.

Secondary objectives of RCT

1. To determine whether HENRY improves parent self-efficacy, eating behaviours, feeding behaviours, dental health, and quality of life
2. To explore whether HENRY influences rates of obesity in parents, siblings and staff (health practitioners)
3. To examine the social and physical environment in the children's centres
4. To monitor any safety issues from the intervention, including adverse events or unintended consequences

Internal pilot objectives of RCT

To assess centre recruitment, parent recruitment and HENRY programme delivery against pre-defined progression criteria.

Economic evaluation objectives

1. To explore the long-term effects on child BMI
2. To determine whether HENRY provides an overall cost saving (e.g. to the NHS)

Process evaluation objectives

1. To produce a map of the system within which HENRY operates and identify hypotheses about how this may be disrupted in response to HENRY
2. To analyse the system in which HENRY is embedded to understand how the system and its elements change over time in response to HENRY
3. To undertake a traditional process evaluation nested within the RCT to understand reach of HENRY within target population, potential contamination and how it has been implemented.

Methods and analysis

Trial design

A multi-centre, open-labelled, two group, prospective, cluster randomised controlled trial, with cost-effectiveness analysis, and embedded mixed methods complex systems evaluation and internal pilot (Figure 1). Eighty two eligible children's centres from within ~14 local authorities (depending on the number of centres per local authority) will be randomly allocated (1:1) to deliver HENRY or continue with standard practice (control). 984 eligible parents will be recruited.

[Figure 1 about here – separate file]

Setting and recruitment

Local authority and centre recruitment

For peer review only - http://bmjopen.bmj.com/site/about/guidelines.xhtml

For peer review only

We plan to recruit local authorities or other associations that are willing to commission HENRY across the UK, from which centres and parents will be recruited. Eligible areas (criteria are detailed below) will then be asked to 1) nominate at least 2 (ideally 6 or more) centres which meet trial eligibility criteria, and 2) sign an agreement before entering the trial. We will record reasons for declining participation, and basic demographic information, from those authorities that choose not to participate.

Local authorities will be asked to nominate approximately twice the number of centres for which they wish to commission HENRY, so that approximately half can be randomised to receive HENRY and half can be randomised to control. Although there are no exclusions based on the demographics of centres, location will be monitored and commissioners encouraged to nominate centres to include a range of diverse social and environmental characteristics. Centre Managers will be given information about the research and asked to provide consent for their centre to participate through a collaboration agreement.

Parent/carer and staff recruitment

Centre staff will invite all parents who are booked to attend a HENRY programme to take part in the research. If interested, they will be screened for eligibility and will be asked to provide informed consent to take part. Those who decline will still be able to attend the HENRY intervention if they wish. Screening and recruitment will aim to start at least 6 weeks prior to the start of each HENRY programme. Programmes typically enrol an average of 8 parents, of whom our design aims to recruit an average of 4 parents. Once consented, blinded data collection staff will contact participants to arrange the baseline visit and complete trial registration. A similar process will recruit parents from the control centres. Here, parents who attend other programmes (e.g. stay and play sessions) will be invited to take part within the same timeframes. Parents will also have the opportunity to self-refer into the trial via recruitment posters displayed in the centres. A £30 shopping voucher will be offered to participants in recognition of their contribution (£15 at baseline, £15 at follow-up). Parent consent will include five options: consent to take part in the trial only, consent to provide height and weight measurements, consent to provide HENRY programme attendance data (if attending a HENRY programme), consent to be contacted about taking part in a process evaluation interview, and consent to share routinely collected National Child Measurement Programme (NCMP) data after 3 years for the purposes of the economic evaluation.

Staff recruitment will occur once they have completed site training. Those agreeing to take part will provide their height, weight and waist circumference, which they can self-measure. Staff will also have the opportunity to consent to be contacted about taking part in a process evaluation interview.

Eligibility criteria

Local authorities can be new to HENRY or already commissioning HENRY, provided they have at least 2 centres (ideally 6) meeting centre eligibility criteria. Local authorities using external teams outside of the centre to deliver HENRY programmes (e.g. health visitors) will be eligible, in addition to those wishing to train internal centre staff to deliver programmes (the most common model). Local authorities without coverage of a National Institute of Health Research (NIHR) local clinical research network (LCRN; the teams responsible for collecting trial data from parents and staff) will not be eligible.

For peer review only - http://bmjopen.bmj.com/site/about/guidelines.xhtml

Centres: Any type of centre or other early years setting such as a nursery or community venue will be eligible to take part provided that they do not already deliver HENRY, and their staff have not received HENRY training (within the past two years). Centres where staff are shared between nominated centres will not be eligible. Centres must aim to run HENRY programmes starting within 12 weeks of training completion and plan to run 3 programmes during the trial. They should be in geographically separate areas to protect against contamination (judged on a case by case basis) and managers must agree to support participant recruitment within their centres. If the centre operates as part of a cluster, that cluster must be deemed to be HENRY naive. HENRY naive clusters are defined as: a group of centres within a cluster that do not include any centres that are (a) currently delivering HENRY or (b) have been trained to, or delivered HENRY within the past 2 years.

Centres that are either currently delivering HENRY or have previously delivered HENRY in the last two years are not eligible – even if they have never delivered HENRY in those centres.

Parents/carers: The target population for the intervention are parents/carers of preschool children. Parents may not be registered more than once but they may be screened on more than one occasion if not registered following first screening, as both eligibility and willingness to participate may change. Parents must have at least 1 child aged 6 months - 5 years (18 months - 6 years at 12 month follow-up) at the time of starting the programme. If more than one child in a family fulfils eligibility criteria, the youngest child (by birth timing if twins) will be considered as the reference child (from which data will be collected). Parents must be willing to attend the programme sessions (intervention centres) and willing to provide data in accordance with the data collection protocol. Parents will be provided with full details of the data collection requirements in advance so that they can make informed decisions as to whether to participate. They must either speak English, or bring their own interpreter with them (e.g. family member) each time they need to respond to questionnaires for trial purposes. Where available, non-English speaking parents will also be able to use local interpreters (provided by the centre or through the local clinical research network). Parents with severe learning difficulties that preclude them taking part in group sessions in which they need to be able to read and write will not be eligible. Those whose reference child is tube fed or with other known clinical conditions likely to affect growth over the period of the trial (e.g. coeliac disease) and those who have attended a HENRY group for a previous child will also be ineligible.

All centre staff will be invited to participate in the research including those directly and indirectly involved in delivering HENRY programmes.

Randomisation and blinding

Following fully signed local authority (and service provider if applicable) and centre agreements, participating centres within each local authority will be randomised to HENRY or control in a 1:1 allocation ratio by the Leeds Clinical Trials Research Unit (CTRU). Minimisation, incorporating a random element, will be used to ensure the treatment groups are well balanced for the following characteristics:

- Size of centre (≤8 / >8 permanent centre members of staff, not including staff using the centre such as Health Visitors, nursery workers etc.,);
- Area level ethnicity (<80% / ≥80% White British using Census data based on centre postcode) and;
- Area level deprivation (≤10% / >10% ranking within Index of Multiple Deprivation at the Lower Layer Super Output Area).

For peer review only - http://bmjopen.bmj.com/site/about/guidelines.xhtml

Parents and centre staff will not be blinded due to the nature of the intervention. Data collection staff will be blinded to centre allocation (and thus the participants' treatment arm).

Active intervention

HENRY was set up with Department of Health funding in 2008 and has been widely commissioned by more than 50 areas (training >15,000 practitioners and providing programmes to ~24,500 parents). It includes core practitioner training and group facilitation training (www.henry.org.uk)(14).

HENRY is delivered primarily within Sure Start centres and other children's centres, as well as other community settings, including schools, mosques and churches. Local authority areas can choose to have HENRY programmes delivered directly by HENRY central teams (less common approach), pay to commission training, licensing and support from HENRY (most common approach), offer a blended approach, or provide training to local staff to deliver programmes (most common in larger local authority areas).

Intervention training for practitioners:

Core practitioner training supports staff to deliver the HENRY approach, which incorporates evidence-based behaviour change models, including the Family Partnership model, motivational interviewing and solution focused support, with information about a healthy start that is consistent with national guidance. This training is designed to allow staff to integrate evidence based models to develop motivation and support lifestyle change for families. This can then be embedded into all interactions with families, in addition to supporting positive changes to the centre environment (space to play, freely available water, food policies etc.). Training is provided to health visitors, dieticians and staff (e.g. at children's centres, community centres/hubs - hereon called 'centres') allowing parental support to be an intrinsic part of their role, whilst influencing culture and policy within early years settings. Training is designed to consistently influence the environment and practice immediately following training.

Facilitator training is delivered to a (usually) smaller selection of staff who have attended core training to certify them to deliver small group sessions 'Right from the Start'. This training enables the delivery of an 8-week universal 'Right from the Start' programme to parents to provide practical skills in authoritative parenting skills, increasing self-esteem, adopting healthy family lifestyles, goal setting, oral health, active play, portion sizes, and learning about food labels.

Training can be delivered in-person or remotely, depending on commissioner and facilitator preference. To allow a richer level of communication, the in-person training can be completed in a shorter time frame (2-day in-person training is delivered over 6 weeks remotely).

Programme delivery to parents:

The 'Right from the Start' programme is delivered to groups of approximately 8-10 parents over 8 sessions. Each interactive session focuses on a separate theme and includes resources for families to take home. Each centre delivers 2-3 'Right from the Start' group programmes per year, each consisting of 8 x 2-hour sessions.

Programmes can be delivered remotely or face-to-face. We will include both delivery modes in the proposed research. This will allow us to be flexible depending on external issues, in addition to supporting an evaluation of a pragmatic intervention. Our analysis plan includes consideration of different delivery approaches.

For peer review only - http://bmjopen.bmj.com/site/about/guidelines.xhtml

Parents with a child aged up to 5 years are eligible to attend HENRY. Language needs are addressed locally depending on the population needs, with some areas providing dedicated support. Without this, the other approach is to invite parents to attend with a friend or family member to support translation and other activities. Although considered to be a Universal programme, practitioners often refer families 'at risk' to HENRY and the delivery model within children's centres allows parents from the most deprived neighbourhoods to attend.

Parents receive a standardised manual as part of the programme. To monitor adherence, session attendance will be recorded for parents who consent.

Control intervention

Staff within control centres will not receive HENRY training and will continue to deliver usual programmes or 'standard practice' (e.g., 'stay and play', 'cook and eat'). In short, this means that families registered to take part in the research will receive the standard level of support provided within their community / centres. Services are aimed at supporting families with a focus on the most disadvantaged families. These vary between and within local areas but usually include access to health visiting teams, breastfeeding support, parenting advice and access to specialist services including speech and language therapy.

Outcomes

The primary outcome for the trial is child age and sex adjusted BMI z-score) assessed at 12 months post parent registration.

Secondary outcomes will be assessed at the same timepoint (defined short-term), after 3 years (medium-term) and will be forecasted across the life-course (longer-term). Outcomes are summarised in Table 1.

For peer review only - http://bmjopen.bmj.com/site/about/guidelines.xhtml

Table 1 Data collection summary

**Data/outcomes**	**Measures**	Collected by	LA[c] / CC[d] Screening and consent	Centre baseline	Participant screening	Participant informed consent and registration	Participant / staff Baseline	Short term follow-up (12 months post registration)	Medium-term follow-up (3 years)[a]	Longer term follow-up[b]
**Local authority**										
Eligibility	LA Eligibility checklist	LA self complete	x							
Basic demographics / reasons for declining	LA / CC Recruitment log	Researcher / HENRY	x							
Centre contact details	Centre nomination form	LA self complete	x							
Local authority agreement / consent	Local authority agreement	LA self complete	x							
**Centre**										
Eligibility	LA Eligibility checklist	LA self complete	x							
Reasons for declining / ineligibility	LA / CC recruitment log	Researcher	x							
Centre agreement / consent	Children's Centre agreement	Centre self complete	x							
Stratification factors	Children's centre	LA self complete		x						

For peer review only - http://bmjopen.bmj.com/site/about/guidelines.xhtml

**Data/outcomes**	**Measures**	Collected by	LA[c] / CC[d] Screening and consent	Centre baseline	Participant screening	Participant informed consent and registration	Participant / staff Baseline	Short term follow-up (12 months post registration)	Medium-term follow-up (3 years)[a]	Longer term follow-up[b]
	nomination form									
Centre randomisation allocation	Randomisation form	CTRU[e]		x						
Demographics	Centre nomination form / Environmental Questionnaire	LA / Centre self complete		x						
Details of other courses	Environmental Questionnaire	Centre self complete		x				x	x	
**Parent**										
Initial eligibility check	Screening form	Centre staff			x					
Confirmation of eligibility	Eligibility checklist	LCRN[f]				x				
Reasons for declining / ineligibility	Screening form	Parent self complete			x					
Demographics (parent, child and family)	Screening form<br>Baseline form	Parent self complete<br>CTRU (recruitment log)			x		x			

For peer review only

**Data/outcomes**	**Measures**	Collected by	LA[c] / CC[d] Screening and consent	Centre baseline	Participant screening	Participant informed consent and registration	Participant / staff Baseline	Short term follow-up (12 months post registration)	Medium-term follow-up (3 years)[a]	Longer term follow-up[b]
Consent to trial participation and data collection (including NCMP)	Consent form	Parent self complete				x				
Use of Centres	Baseline form<br>Follow up form	LCRN					x	x	x	
**Outcomes**										
Child age and sex adjusted BMI Z-score, child height, child weight, unadjusted BMI and weight/BMI percentiles	Measured	LCRN (baseline & 12 M follow-up)<br><br>Longer term: CTRU accessing routine data					x	x	x	
Sibling age and sex adjusted BMI, height (m), weight (kg), unadjusted BMI and weight/BMI percentiles		CTRU accessing routine data							x	
Parent self-efficacy	Dumka (27)	LCRN					x	x		
Family eating / activities	Golan (28)	LCRN					x	x		
Feeding questionnaire	Baughcum (29)	LCRN					x	x		

For peer review only

**Data/outcomes**	**Measures**	Collected by	LA[c] / CC[d] Screening and consent	Centre baseline	Participant screening	Participant informed consent and registration	Participant / staff Baseline	Short term follow-up (12 months post registration)	Medium-term follow-up (3 years)[a]	Longer term follow-up[b]
Dental health (child)	Dental questionnaire	LCRN					x	x		
Parent height & weight:	Measured	LCRN					x	x		
Parent waist circumference	Measured	LCRN					x	x		
**Staff:**										
Staff screening	Staff screening form	Staff self complete at centre		x						
Staff height & weight	Measured	Self-measure					x	x		
Staff waist circumference	Measured	Self-measure					X	x		
**Children's Centre:**										
Centre social, physical and political environment (e.g. policies around food)	Environmental questionnaire	Centre self complete		x				x		
**Safety:**										

For peer review only

For peer review only - http://bmjopen.bmj.com/site/about/guidelines.xhtml

**Data/outcomes**	**Measures**	Collected by	LA[c] / CC[d] Screening and consent	Centre baseline	Participant screening	Participant informed consent and registration	Participant / staff Baseline	Short term follow-up (12 months post registration)	Medium-term follow-up (3 years)[a]	Longer term follow-up[b]
Adverse events / unintended consequences	SAE / RUSAE forms	LCRN / centre self complete						x		
**Health economics:**										
Health care resource use for child	Resource use questionnaire	LCRN					x	x		
Health care resource use for parents	Resource use questionnaire	LCRN					x	x		
Private costs (e.g. travel costs, additional food expenses, lost productivity)	Resource use questionnaire	LCRN					x	x		
Parent's Health-related Quality of Life	EQ-5D-5L (30) and ICECAP-A [31]	LCRN					x	x		
**Routine data:**										
NCMP Child and sibling data (trial participants)		CTRU							x	
NCMP regional child data (not trial participants)		CTRU							x	

For peer review only

**Data/outcomes**	**Measures**	Collected by	LA[c] / CC[d] Screening and consent	Centre baseline	Participant screening	Participant informed consent and registration	Participant / staff Baseline	Short term follow-up (12 months post registration)	Medium-term follow-up (3 years)[a]	Longer term follow-up[b]
Process evaluation/delivery of HENRY programmes:										
Attendance * *Data collection not timed to trial time points	HENRY attendance records	HENRY Centre staff / HENRY facilitator								
Contamination checks* *Data collection not timed to trial time points	Staff movement form	Staff self complete								
HENRY training attendance and knowledge		HENRY						x	x	
**Qualitative data**										
System map and sub maps* *Data collection not timed to trial time points	n/a	Researcher								
Systems mapping interviews with local authority stakeholders* *Data collection not timed to trial time points	n/a	Researcher								

For peer review only

For peer review only - http://bmjopen.bmj.com/site/about/guidelines.xhtml

**Data/outcomes**	**Measures**	Collected by	LA[c] / CC[d] Screening and consent	Centre baseline	Participant screening	Participant informed consent and registration	Participant / staff Baseline	Short term follow-up (12 months post registration)	Medium-term follow-up (3 years)[a]	Longer term follow-up[b]
Interviews with centre staff* *Data collection not timed to trial time points	n/a	Researcher								
Interviews with parents* **Data collection not timed to trial time points	n/a	Researcher								

[a] Medium term outcomes will gathered from routinely collected data (from health visitors and/or the National Child Measurement Programme)

[b] Longer term outcomes will be based on matched cohorts of (Millennium Cohort Study (MCS ((15)).

[C] Local Authority (or other governing/commissioning body)

[d] Children's centre (or other setting in which HENRY can be delivered, including Sure Start centres in Northern Ireland)

[e] Clinical Trials Research Unit

[f] Local Clinical Research Network (England only). Other devolved nations will train researchers/equivalent staff to collect data)

For peer review only

Data collection

Table 1 provides information about the types of data collected and how these are collected. Baseline and follow-up data (12 months) will be collected within participant homes by trained Local Clinical Research Network (LCRN) staff (or equivalent trained staff in the devolved nations). The majority of questionnaires completed during home visits will be interviewer administered. Centre level outcomes will be completed by a nominated member of centre staff (usually the centre manager or HENRY facilitator). Staff level data will be self-completed and entered directly on to the online database.

Sample size

41 centres per arm (from 10-14 local authorities), each recruiting 12 parents on average (4 parents from 3 programmes, 984 parents in total) will provide 90% power to detect a small standardised effect size of 0.27 as per previous trials [25-28] for BMI z-score at a 5% significance level, assuming an ICC of 0.03 [16, 25, 29-31] to account for clustering by centre, a coefficient of variation of 0.48 to account for variation in centre recruitment and 20% loss to follow-up (16).

Withdrawal of consent

Centres and/or local authorities can withdraw at any point during the trial. Data collected up to the time of withdrawal will be retained for analysis and data from parents and staff will still be collected provided they have not withdrawn consent themselves. Centres may stop delivering the HENRY programme during the trial period independently from the trial (e.g. centre closures, restructuring). Trial procedures will continue in this eventuality and all recruited parents and staff will remain in the trial (and data will continue to be collected from them) unless they actively withdraw. Where parents or staff wish to withdraw, there will be clarification whether this is withdrawal from short term trial data collection, or from medium term trial data collection (using routine data) or a combination of these. Non-attendance at HENRY intervention sessions are not classed as a withdrawal from the trial. All parents who withdraw from HENRY intervention or who do not attend the intervention will still be followed–up for data collection unless they specifically express a wish to withdraw from trial processes.

Data analysis plan

Analysis will be carried out on the intention-to-treat (ITT) population defined as all local authorities / centres randomised and all parents registered to the trial, regardless of adherence to the protocol, withdrawal of consent or losses to follow-up. A two-sided 5% significance level will be used for statistical endpoint comparisons. No interim analyses are planned, except for safety data that are required for review by the Data Monitoring and Ethics Committee.

The flow of local authorities, centres and participants through the trial will be presented in a Consolidated Standards of Reporting Trials diagram.

The primary outcome, child age and sex adjusted BMI (BMI z-score), will be analysed using a multi-level linear regression with children nested within centres, and centres treated as a random effect. The model will be adjusted for the following fixed effects: centre-level stratification factors, important parent-level and child-level covariates (e.g. baseline child BMI z-score and sex, parent BMI), and other relevant known predictors of outcome. Missing data will be imputed at the individual participant level where appropriate. Estimated mean differences will be reported with

For peer review only - http://bmjopen.bmj.com/site/about/guidelines.xhtml

95% confidence intervals, p-values and ICCs. Model diagnostics will be used to check the underlying assumptions of the model and alternative methodology will be used if required.

Sensitivity analyses of the primary endpoint will be conducted to assess the impact of missing data, the choice of imputation model and the missing at random assumption, as appropriate. If contamination between intervention and control centres is identified, a sensitivity analysis excluding the relevant control centres will be conducted.

If numbers allow, exploratory subgroup analysis will examine differences in intervention effect between different socio-economic and ethnic groups, and also between online and face-to-face delivery of HENRY.

For secondary outcomes, summary statistics will be presented at baseline and 12 months post-parent registration overall and by arm (means, standard deviations, medians, minimum, maximum, and quartiles for continuous variables, and counts and percentages for categorical variables).

Medium-term analysis (3-years post parent recruitment) will compare regional population level BMI z-score (trial local authority areas) with that of trial participants and siblings (HENRY and control) to investigate differences in BMI z-score. Analysis will use the same approach as the primary outcome for different outcome types, using multi-level regression with multiple imputation for missing data.

Internal pilot

Descriptive analysis of the internal pilot against progression criteria will take place at three separate time points as described in Table 2 and discussed with the trial steering committee to inform a decision on the modification or continuation of the trial.

Table 2. Internal pilot progression criteria

Criteria	Green (go)	Amber (review)	Red (stop)
**Centre Recruitment**<br>Centres open within 12 months of starting centre recruitment<br>(including data up to the end of month 19). A centre will be defined as open if it has been randomised and is open to recruitment.	≥54	42-53	<42
**Parent Recruitment**<br>Average number of parents recruited per programme / equivalent<br>(including data up to the end of month 23, allowing for 6 months of parent recruitment).	≥4	3 to <4	<3
**HENRY Programme delivery**<br>Percentage of intervention centres having started delivery of at least 1 programme<br>(including data up to the end of month 27, allowing for 18 months from starting centre recruitment). The denominator will include all centres allocated to the intervention arm. The numerator will be all of those intervention centres who have started to deliver at least one programme,	≥80%	50% to 80%	<50%

For peer review only - http://bmjopen.bmj.com/site/about/guidelines.xhtml

Criteria	Green (go)	Amber (review)	Red (stop)
defined as delivering at least one session to parents.			

Economic evaluation

Economic analysis will be conducted in three stages: (1) 12 month short-term within-trial cost-effectiveness analysis of the incremental cost per unit change in BMI z-score; (2) Three year cost-effectiveness analysis of the incremental cost per unit change in BMI z-score with additional routinely collected National Child Measurement Programme data; and (3) Longer-term estimates of BMI z-score trajectories and healthcare use using a matched cohort of Millennium Cohort Study (MCS) participants (15).

Primary analyses will adopt an NHS and local authority perspective. Supplementary analyses will adopt a wider societal perspective by assessing household costs and productivity losses. Spill over benefits to parents will also be captured by calculating differences between the two treatment groups in terms of quality adjusted life years (QALYs) over 12 months. Utility scores will be measured using both the EQ-5D-5L (17) and the ICECAP-A (18). All analyses will be conducted using the ITT population. Seemingly unrelated regression will be used to account for the correlation between costs and outcome measures. Multilevel models will be used to account for children nested within centres. Decisions about which child-level, parent-level and centre-level covariates to include in the models will be made after assessing differences in baseline characteristics, and through discussion with the trial statisticians. Patterns (and reasons) of missing data will be investigated in collaboration with the trial statisticians and appropriate imputation techniques will be used. Supplementary analyses will assess whether there are differences in costs and effectiveness by the intervention delivery method by including an interaction term between method (i.e., online vs. face-to-face) and the treatment variable. In order to address whether or not the intervention is equally cost-effective among children living in the most deprived areas and households, analyses will also use interaction terms for household-level socioeconomic status and for area level deprivation (≤ 10%/> 10% ranking within Index of Multiple Deprivation at the Lower Super Output Area). To assess whether a parent/carer's propensity to commit to engage with HENRY was affected by their attitude to risk or perceptions about their long-term health prospects, analyses will also use interaction terms for self-reported measures of these factors based on questions used in the English Longitudinal Study of Ageing (ELSA) (19) and German Socioeconomic Panel (SOEP) (20). Uncertainty in our cost-effectiveness estimates will be characterised by presenting bootstrapped estimates on CEACs using a wide range of different cost-effectiveness thresholds (£/BMI change). Results for all economic analyses will be reported in accordance with the Consolidated Health Economic Evaluation Reporting Standards (CHEERS) guidelines (21).

Systems-based process evaluation

HENRY is a complex intervention that can be viewed as an event within a system (22). Therefore, we will adopt a complex systems perspective, using a framework for qualitative systems process evaluations (23) and quantitative systems evaluation, embedded within a mixed methods process evaluation (24). The evaluation will consist of the two-stage qualitative systems approach: (1) initial systems mapping, and (2) analysis of the system within which HENRY is embedded. It will also include a traditional process evaluation to understand context, mechanisms and implementation of HENRY (24) and quantitative systems modelling.

For peer review only - http://bmjopen.bmj.com/site/about/guidelines.xhtml

The systems map will be constructed using data collected at a stakeholder workshop (n=30-40) at the start of the RCT in which we will identify: structure (e.g. levels of national, regional and local); elements (e.g. national public health priorities around childhood health, obesity and parenting; local authority priorities and funding situation; health visitor responsibilities; organisations providing HENRY; welfare benefits systems; historical events affecting childhood obesity); relationships and interactions between elements; and boundaries (what is inside and outside the system). The overall map may ultimately consist of a number of sub-maps that will highlight causal inter-relationships between each of the elements of these areas. It may also present HENRY at different levels (the local authority, centre, parent/child and other) identified via this research. These maps will comprise fundamental systems thinking 'building blocks' in the form of causal-loop diagrams and/or stock-and-flow structures as appropriate. Each set of diagrams (or system maps) will seek to relate parameter/variable components of the local system through positive/negative causality and ordinalities such that a dynamic representation of inter-relationships can be viewed graphically.

The second stage of qualitative systems process evaluation will explore how the system and its elements change over time in response to HENRY. Six months prior to the end of the RCT, we will undertake qualitative telephone interviews/virtual interviews with 20-24 national, regional and local key stakeholders representing elements of the Stage 1 map to discuss the system structure and elements, how they have changed over time, the way the system responded to HENRY, and how responses amplified or dampened HENRY's impacts. We will also explore how HENRY affected or was affected by potential strategies for addressing health inequalities.

To complement McGill's qualitative systems process evaluation, we will also focus on understanding the reach of HENRY within the target population, how HENRY works, potential contamination, and how it has been implemented within the RCT (24). We will undertake semi-structured interviews with staff providing HENRY (n=12-16) to explore their views of the feasibility of HENRY, how it has been delivered in different centres over time (variation in implementation), access to HENRY for different socio-economic and ethnic minority groups (reach), perceptions of differential health outcomes for different socio-economic and ethnic minority groups (that is, how it addresses health inequalities or not), and views of potential contamination in the RCT. We will also undertake semi-structured interviews with parents (n=20-24) attending HENRY to explore acceptability of the intervention and perceptions of how it facilitated health improvement or not. In both sets of interviews, we will explore mechanisms of impact (how HENRY has affected parenting, nutrition and weight of children), facilitators and barriers to delivering or attending HENRY, reach in terms of accessing those most in need, and implementation in practice. We will also include a systems lens by putting context at the centre of the interviews and explore the impact of system elements on HENRY, specifically asking about how elements of the system documented in Stage 1 interacted with HENRY. These interviews may affect our understanding of why HENRY was implemented in different ways in different localities or at different times, and the extent to which health inequalities have been addressed by HENRY.

Quantitative process data will be gathered to summarise attendance and drop out by centre and overall and will support the monitoring and evaluation of contamination. Thus, in addition to exploring contamination through qualitative interviews, data on staff movement and sharing of HENRY related messages will be captured. Key to this will be an online HENRY-trained staff survey at two time periods (towards the end of the internal pilot and at the end of the RCT intervention period) asking whether, how and when staff shared HENRY practice outside their centre.

Analysis of qualitative data will use the Framework approach, developing an initial thematic framework based on familiarisation of a range of transcripts, analysis of qualitative interviews from

For peer review only - http://bmjopen.bmj.com/site/about/guidelines.xhtml

the feasibility study (25), and the process evaluation framework (mechanisms, context and implementation) (24). Quantitative evaluation will combine trial and process data to apply a causality-based model of obesity prevention to develop a systems archetype (26). Analysis will incorporate Morphological Analysis and Fuzzy Cognitive Mapping techniques (27) to evaluate and assign inter-relationships between components through causal weights and network directionality. We will examine the range of operating conditions of the HENRY system model to classify its overall dynamic behaviour (identifying which Systems Archetype HENRY most closely resembles (26); providing a basis for comparison to other obesity models (e.g., Foresight) to explain how HENRY may disrupt obesity prevalence in childhood).

Public involvement

We will consult closely with a diverse group of parents with young children from a local children's centre. Activities will include workshop-style meetings timed to project milestones and in line with trial steering committee meetings. Each workshop will have a specific aim and associated set of activities including brainstorming and group discussion. We will share workshop outcomes with research team members, including the steering committee, and, where applicable, incorporate them in research activities. To begin, the group will be asked to consider their needs for involvement, including training or practical requirements. The dedicated public involvement lead (WB) will be responsible for setting and overseeing the overall strategy. Proposed activities will be embedded in all stages of the research. For example, during recruitment, we will discuss unanticipated barriers (e.g., Covid related issues) so that we can help mitigate these. The group will provide input into the process evaluation work; for example, ensuring concepts and language used during systems mapping workshops is appropriate. They will also be involved in interpreting trial results in a way most relevant to parents and help to design and disseminate trial outputs.

Ethics and dissemination

The trial will adhere to ethical principles, approach, aims and methods of the ESRC research framework. Ethical approval was obtained by the University of York, Health Sciences' Research Governance Committee (HSRGC/2022/537/E). HRA approval was also obtained to allow NHS (e.g. children's centres sitting within integrated care organisations) sites to take part in the trial (Integrated Research Application System (ID:317992). Serious adverse events are not anticipated; however, we have convened an independent trial steering committee (TSC) and separate data monitoring committee (DMEC) to monitor trial progress and adherence (TSC) and ethical issues and safety (DMEC).

Our dissemination strategy includes both the production of regular progress reports and outputs to share with local and national governments, and final reports and publications. For example, we will disseminate findings of the systems work via meetings and conferences, and via publications (i.e., not restricted to the end of trial). Our strategy also includes regular public facing newsletters, social media outputs and attendance at festival events. Final outputs will be shared via a dissemination event, policy briefings and academic publications/presentations.

Discussion

This is a timely opportunity to carry out a detailed evaluation of an obesity prevention programme that has been delivered at scale for over 10 years. The findings have the potential to inform and shape the future commissioning landscape, in addition to considering the wider obesity strategies within local authority areas. The long-term commitment to invest in public health and prevention, particularly related to obesity and obesity-related diseases requires an investment in evaluation

For peer review only - http://bmjopen.bmj.com/site/about/guidelines.xhtml

evidence to ensure that funding is best used to ensure optimal reach and impact, particularly to those living in the most deprived areas. Specifically, the findings will provide (1) the effectiveness of an intervention delivered at scale to support commissioning decision making; (2) a robust estimation of the cost effectiveness associated with the programme based on obesity at 12 months, in addition to long-term impact after approximately 3 years and modelling of longer-term impact (from the perspective of the NHS combining additional routine data from the NCMP); (3) an understanding of the role that HENRY plays to disrupt the wider obesity prevention system within local authority areas to support national and local obesity strategies, and enable continued monitoring and evaluation; (4) an understanding of the mechanisms of impact of HENRY on obesity, parenting and behaviour, including the facilitators and barriers to delivering or attending HENRY, its reach in terms of accessing those most in need, and implementation in practice; and (5) an understanding of patients' and staffs' views and experiences of HENRY, including feasibility and acceptability to support implementation planning.

Author contributions

MB: Conceptualisation, Funding acquisition, Oversight (study lead), Methodology, Writing- Original draft, Writing- Reviewing and Editing; **WB**: Conceptualisation, Funding acquisition, project management, PPI lead, Writing- Original draft, Writing- Reviewing and Editing; **MC**: Funding acquisition, Methodology (statistical lead), Writing- Original draft, Writing- Reviewing and Editing; **AM**: Funding acquisition, Methodology (economics lead), Writing- Original draft, Writing- Reviewing and Editing; **BC**: Funding acquisition, Methodology (analysis), Writing- Original draft, Writing- Reviewing and Editing; **DGW:** Methodology (Trial management), Writing- Original draft, Writing- Reviewing and Editing; **AF**: Methodology (systems process evaluation), Writing- Original draft, Writing- Reviewing and Editing; **TW**: Methodology (Trial operations), Writing- Original draft, Writing- Reviewing and Editing; **AS:** Funding acquisition, Methodology (systems lead), Writing- Original draft, Writing- Reviewing and Editing; **AOC:** Funding acquisition, Methodology (systems process evaluation lead), Writing- Original draft, Writing- Reviewing and Editing;

Competing interests

No authors report any conflicts of interest

Acknowledgements

Funding

This study/project is funded by the NIHR [name of NIHR programme (NIHR135081]. The views expressed are those of the authors and not necessarily those of the NIHR or the Department of Health and Social Care.

Public involvement

We acknowledge our parent advisory group for their support in developing the intervention and ongoing advice in study design and recruitment, including Lauren Surtees, Sarah Green, Abeer Mukhta and Lina Alrefaai.

Trial Steering Committee (TSC) /Data Monitoring Committee (DMEC)

We thank the members of the TSC, including Professor Peymane Adab (TSC Chair, University of Birmingham), Professor Amelia Lake (Teeside University), Professor Laura Cornelsen (London School

For peer review only - http://bmjopen.bmj.com/site/about/guidelines.xhtml

of Hygiene and Tropical Medicine), Dr. Nasima Akhter (Teeside University) and Lina Alrefaai (parent representative), Paul Atkin (CRN Yorkshire), Professor Simon Coulton (University of Kent), Lauren Surtees (parent representative). We are also grateful to our DMEC members, Dr Sarah Seaton (University of Leicester), Professor Miranda Pallan (University of Birmingham) and Dr Sarah Crozier (University of Southampton).

References

1. NHS Digital. The National Child Measurement Programme England 2020/21 School Year 2021 https://digital.nhs.uk/data-and-information/publications/statistical/national-child-measurement-programme/2020-21-school-year. Accessed 21.11.22
2. Browne NT, Snethen JA, Greenberg CS, Frenn M, Kilanowski JF, Gance-Cleveland B, et al. When Pandemics Collide: The Impact of COVID-19 on Childhood Obesity. J Pediatr Nurs. 2021;56:90-8.
3. Hasan T, Ainscough TS, West J, Fraser LK. Healthcare utilisation in overweight and obese children: a systematic review and meta-analysis. BMJ open. 2020;10(10):e035676.
4. Sharma V, Coleman S, Nixon J, Sharples L, Shield J, Rutter H, et al. Systematic Review and Meta-Analysis of Co-morbidities Associated with Child and Adolescent Obesity (aged 5-18). Obesity Reviews. 2019;20:1341-9.
5. Pandita A, Sharma D, Pandita D, Pawar S, Tariq M, Kaul A. Childhood obesity: prevention is better than cure. Diabetes Metab Syndr Obes. 2016;9:83-9.
6. Public Health England. PHE Strategy 2020-25. 2019 Sept 2019. https://assets.publishing.service.gov.uk/government/uploads/system/uploads/attachment_data/file/830105/PHE_Strategy__2020-25__Executive_Summary.pdf Accessed
7. Redsell S, Atkinson P, Nathan D, Siriwardena N, Swift J, Glazebrook C. Primary prevention of childhood obesity: views from primary care. Archives of Disease in Childhood. 2011;96(Suppl 1):A9-A11.
8. Redsell SA, Edmonds B, Swift JA, Siriwardena AN, Weng S, Nathan D, et al. Systematic review of randomised controlled trials of interventions that aim to reduce the risk, either directly or indirectly, of overweight and obesity in infancy and early childhood. Maternal & child nutrition. 2016;12(1):24-38.
9. Hillier F, Pedley C, Summerbell C. Evidence base for primary prevention of obesity in children and adolescents. Bundesgesundheitsblatt, Gesundheitsforschung, Gesundheitsschutz. 2011;54(3):259-64.
10. Brown T, Moore THM, Hooper L, Gao Y, Zayegh A, Ijaz S, et al. Interventions for preventing obesity in children. Cochrane Database of Systematic Reviews. 2019(7).
11. Carey G, Malbon E, Carey N, Joyce A, Crammond B, Carey A. Systems science and systems thinking for public health: a systematic review of the field. BMJ open. 2015;5(12):e009002.
12. Owen L, Morgan A, Fischer A, Ellis S, Hoy A, Kelly MP. The cost-effectiveness of public health interventions. Journal of Public Health. 2011;34(1):37-45.
13. Bryant M, Burton W, Collinson M, Hartley S, Tubeuf S, Roberts K, et al. Cluster randomised controlled feasibility study of HENRY: a community-based intervention aimed at reducing obesity rates in preschool children. Pilot and Feasibility Studies. 2018;4(1):118.
14. Willis TA, Potrata B, Hunt C, Rudolf MC. Training community practitioners to work more effectively with parents to prevent childhood obesity: the impact of HENRY upon Children's Centres and their staff. Journal of human nutrition and dietetics : the official journal of the British Dietetic Association. 2012;25(5):460-8.
15. Connelly R, Platt L. Cohort Profile: UK Millennium Cohort Study (MCS). International Journal of Epidemiology. 2014;43(6):1719-25.

For peer review only - http://bmjopen.bmj.com/site/about/guidelines.xhtml

16. Bryant M, Burton W, Collinson M, Farrin A, Nixon J, Stevens J, et al. A cluster RCT and process evaluation of an implementation optimisation intervention to promote parental engagement enrolment and attendance in a childhood obesity prevention programme: results of the Optimising Family Engagement in HENRY (OFTEN) trial. Trials. 2021;22(1):773.
17. Devlin NJ, Shah KK, Feng Y, Mulhern B, van Hout B. Valuing health-related quality of life: An EQ-5D-5L value set for England. Health economics. 2018;27(1):7-22.
18. Al-Janabi H, N Flynn T, Coast J. Development of a self-report measure of capability wellbeing for adults: the ICECAP-A. Quality of Life Research. 2012;21(1):167-76.
19. Minicuci N, Naidoo N, Corso B, Rocco I, Chatterji S, Kowal P. Data Resource Profile: Cross-national and cross-study sociodemographic and health-related harmonized domains from SAGE plus CHARLS, ELSA, HRS, LASI and SHARE (SAGE+ Wave 2). Int J Epidemiol. 2019;48(1):14-j.
20. Wagner GG, Frick JR, Schupp J, Berlin J. The German Socio-Economic Panel Study (SOEP): Scope, Evolution and Enhancements, SOEPpapers on Multidisciplinary Panel Data Research, No. 1. Deutsches Institut für Wirtschaftsforschung (DIW)2007.
21. Husereau D, Drummond M, Petrou S, Carswell C, Moher D, Greenberg D, et al. Consolidated Health Economic Evaluation Reporting Standards (CHEERS) statement. BMJ : British Medical Journal. 2013;346:f1049.
22. Hawe P, Shiell A, Riley T. Theorising interventions as events in systems. Am J Community Psychol. 2009;43(3-4):267-76.
23. McGill E, Marks D, Er V, Penney T, Petticrew M, Egan M. Qualitative process evaluation from a complex systems perspective: A systematic review and framework for public health evaluators. PLOS Medicine. 2020;17(11):e1003368.
24. Moore G, Audrey S, Barker M, Bond L, Bonell C, Hardeman W, et al. Process evaluation of complex interventions: Medical Research Council guidance. BMJ. 2015;350(h1258).
25. Ritchie J, Spencer L. The Qualitative Researcher's Companion. 2002 2023/10/27. Thousand Oaks

Thousand Oaks, California: SAGE Publications, Inc. Available from: https://methods.sagepub.com/book/the-qualitative-researchers-companion.
26. Braun W. The System Archetypes: The Systems Modeling Workbook2002 16.07.21.
27. Sharif A., Irani Z. Fuzzy Cognitive Mapping for MIS Decision-Making. . In: (eds) KC, editor. Fuzzy Applications in Industrial Engineering. 201. Berlin, Heidelberg: Springer; 2006.

For peer review only

FIGURE 1: TRIAL SUMMARY

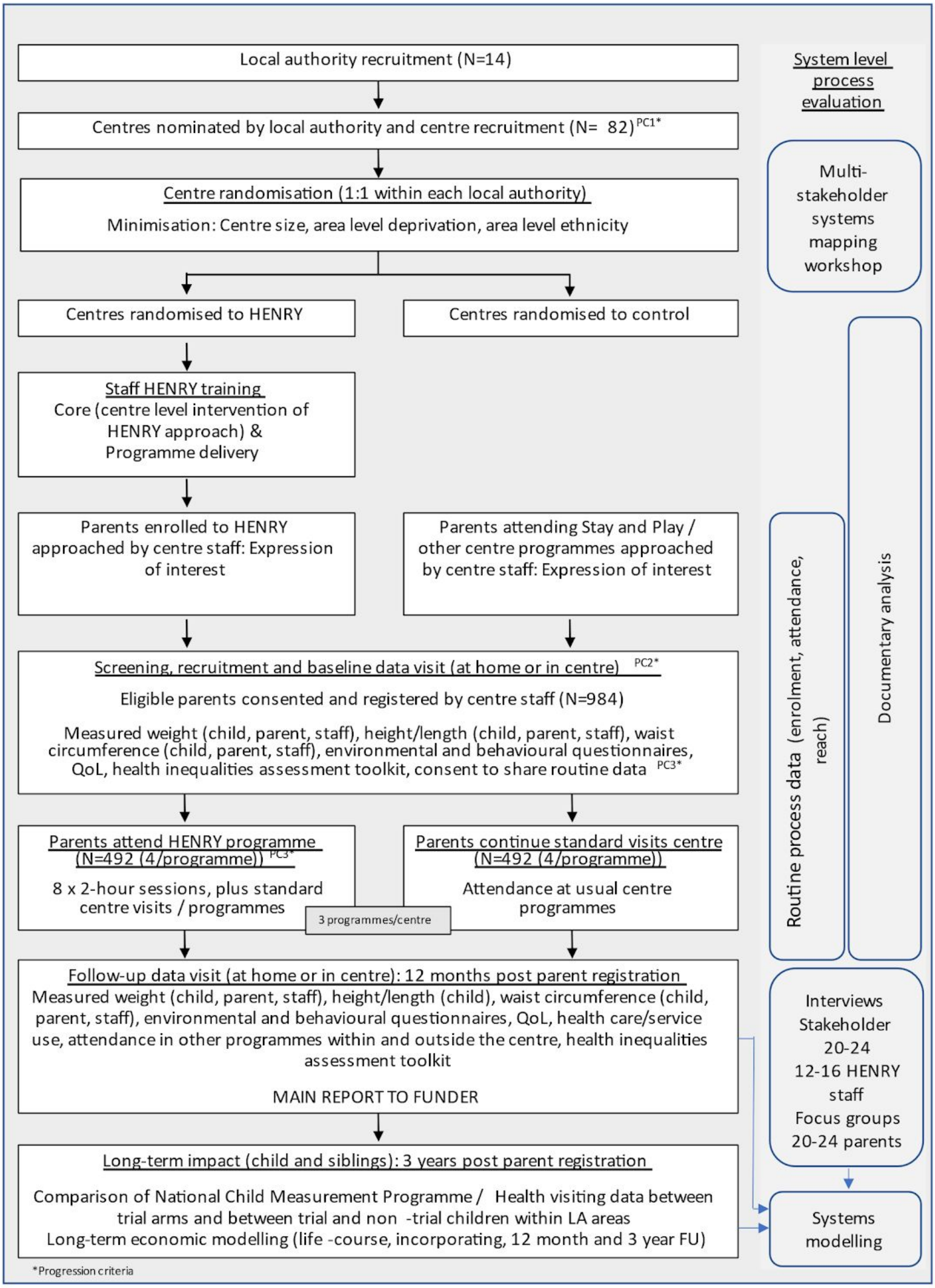

STANDARD PROTOCOL ITEMS: RECOMMENDATIONS FOR INTERVENTIONAL TRIALS

SPIRIT 2013 Checklist: Recommended items to address in a clinical trial protocol and related documents*

NB: Items with a ‘RP’ response are available on the registered protocol (ISRCTN16529380)

Section/item	Item No	Description	Addressed on page number
**Administrative information**			
Title	1	Descriptive title identifying the study design, population, interventions, and, if applicable, trial acronym	___1___________
Trial registration	2a	Trial identifier and registry name. If not yet registered, name of intended registry	___2___________
	2b	All items from the World Health Organization Trial Registration Data Set	______________
Protocol version	3	Date and version identifier	__RP_________
Funding	4	Sources and types of financial, material, and other support	___6___________
Roles and responsibilities	5a	Names, affiliations, and roles of protocol contributors	___1,6_________
	5b	Name and contact information for the trial sponsor	___RP__________
	5c	Role of study sponsor and funders, if any, in study design; collection, management, analysis, and interpretation of data; writing of the report; and the decision to submit the report for publication, including whether they will have ultimate authority over any of these activities	___RP_______

For peer review only

	5d	Composition, roles, and responsibilities of the coordinating centre, steering committee, endpoint adjudication committee, data management team, and other individuals or groups overseeing the trial, if applicable (see Item 21a for data monitoring committee)	___RP______
**Introduction**			
Background and rationale	6a	Description of research question and justification for undertaking the trial, including summary of relevant studies (published and unpublished) examining benefits and harms for each intervention	___4,5________
	6b	Explanation for choice of comparators	____9_________
Objectives	7	Specific objectives or hypotheses	____4,5________
Trial design	8	Description of trial design including type of trial (eg, parallel group, crossover, factorial, single group), allocation ratio, and framework (eg, superiority, equivalence, noninferiority, exploratory)	____5_________
**Methods: Participants, interventions, and outcomes**			
Study setting	9	Description of study settings (eg, community clinic, academic hospital) and list of countries where data will be collected. Reference to where list of study sites can be obtained	____5,6________
Eligibility criteria	10	Inclusion and exclusion criteria for participants. If applicable, eligibility criteria for study centres and individuals who will perform the interventions (eg, surgeons, psychotherapists)	____6_________
Interventions	11a	Interventions for each group with sufficient detail to allow replication, including how and when they will be administered	____8,9________
	11b	Criteria for discontinuing or modifying allocated interventions for a given trial participant (eg, drug dose change in response to harms, participant request, or improving/worsening disease)	____NA________
	11c	Strategies to improve adherence to intervention protocols, and any procedures for monitoring adherence (eg, drug tablet return, laboratory tests)	____20________
	11d	Relevant concomitant care and interventions that are permitted or prohibited during the trial	____NA______

For peer review only - http://bmjopen.bmj.com/site/about/guidelines.xhtml

Outcomes	12	Primary, secondary, and other outcomes, including the specific measurement variable (eg, systolic blood pressure), analysis metric (eg, change from baseline, final value, time to event), method of aggregation (eg, median, proportion), and time point for each outcome. Explanation of the clinical relevance of chosen efficacy and harm outcomes is strongly recommended	___9-16______
Participant timeline	13	Time schedule of enrolment, interventions (including any run-ins and washouts), assessments, and visits for participants. A schematic diagram is highly recommended (see Figure)	___Figure 1_____
Sample size	14	Estimated number of participants needed to achieve study objectives and how it was determined, including clinical and statistical assumptions supporting any sample size calculations	___17____
Recruitment	15	Strategies for achieving adequate participant enrolment to reach target sample size	___6-7________

Methods: Assignment of interventions (for controlled trials)

Allocation:

Sequence generation	16a	Method of generating the allocation sequence (eg, computer-generated random numbers), and list of any factors for stratification. To reduce predictability of a random sequence, details of any planned restriction (eg, blocking) should be provided in a separate document that is unavailable to those who enrol participants or assign interventions	___7________
Allocation concealment mechanism	16b	Mechanism of implementing the allocation sequence (eg, central telephone; sequentially numbered, opaque, sealed envelopes), describing any steps to conceal the sequence until interventions are assigned	____RP________
Implementation	16c	Who will generate the allocation sequence, who will enrol participants, and who will assign participants to interventions	____RP________
Blinding (masking)	17a	Who will be blinded after assignment to interventions (eg, trial participants, care providers, outcome assessors, data analysts), and how	____7-8________
	17b	If blinded, circumstances under which unblinding is permissible, and procedure for revealing a participant's allocated intervention during the trial	____RP________

Methods: Data collection, management, and analysis

Data collection methods	18a	Plans for assessment and collection of outcome, baseline, and other trial data, including any related processes to promote data quality (eg, duplicate measurements, training of assessors) and a description of study instruments (eg, questionnaires, laboratory tests) along with their reliability and validity, if known. Reference to where data collection forms can be found, if not in the protocol	___17 & Table 1_
	18b	Plans to promote participant retention and complete follow-up, including list of any outcome data to be collected for participants who discontinue or deviate from intervention protocols	____17_______
Data management	19	Plans for data entry, coding, security, and storage, including any related processes to promote data quality (eg, double data entry; range checks for data values). Reference to where details of data management procedures can be found, if not in the protocol	__RP____
Statistical methods	20a	Statistical methods for analysing primary and secondary outcomes. Reference to where other details of the statistical analysis plan can be found, if not in the protocol	___17-18_____
	20b	Methods for any additional analyses (eg, subgroup and adjusted analyses)	__18__
	20c	Definition of analysis population relating to protocol non-adherence (eg, as randomised analysis), and any statistical methods to handle missing data (eg, multiple imputation)	__17________

Methods: Monitoring

Data monitoring	21a	Composition of data monitoring committee (DMC); summary of its role and reporting structure; statement of whether it is independent from the sponsor and competing interests; and reference to where further details about its charter can be found, if not in the protocol. Alternatively, an explanation of why a DMC is not needed	__21-22______
	21b	Description of any interim analyses and stopping guidelines, including who will have access to these interim results and make the final decision to terminate the trial	___17________
Harms	22	Plans for collecting, assessing, reporting, and managing solicited and spontaneously reported adverse events and other unintended effects of trial interventions or trial conduct	__21 & Table 1_
Auditing	23	Frequency and procedures for auditing trial conduct, if any, and whether the process will be independent from investigators and the sponsor	___21________

Ethics and dissemination

For peer review only - http://bmjopen.bmj.com/site/about/guidelines.xhtml

Research ethics approval	24	Plans for seeking research ethics committee/institutional review board (REC/IRB) approval	___2________
Protocol amendments	25	Plans for communicating important protocol modifications (eg, changes to eligibility criteria, outcomes, analyses) to relevant parties (eg, investigators, REC/IRBs, trial participants, trial registries, journals, regulators)	__RP_____
Consent or assent	26a	Who will obtain informed consent or assent from potential trial participants or authorised surrogates, and how (see Item 32)	__6 & Table 1_
	26b	Additional consent provisions for collection and use of participant data and biological specimens in ancillary studies, if applicable	____NA______
Confidentiality	27	How personal information about potential and enrolled participants will be collected, shared, and maintained in order to protect confidentiality before, during, and after the trial	_____RP_____
Declaration of interests	28	Financial and other competing interests for principal investigators for the overall trial and each study site	_____22_____
Access to data	29	Statement of who will have access to the final trial dataset, and disclosure of contractual agreements that limit such access for investigators	____RP_______
Ancillary and post-trial care	30	Provisions, if any, for ancillary and post-trial care, and for compensation to those who suffer harm from trial participation	____NA________
Dissemination policy	31a	Plans for investigators and sponsor to communicate trial results to participants, healthcare professionals, the public, and other relevant groups (eg, via publication, reporting in results databases, or other data sharing arrangements), including any publication restrictions	____21_______
	31b	Authorship eligibility guidelines and any intended use of professional writers	___RP________
	31c	Plans, if any, for granting public access to the full protocol, participant-level dataset, and statistical code	___NA________
**Appendices**			
Informed consent materials	32	Model consent form and other related documentation given to participants and authorised surrogates	___RP________

For peer review only - http://bmjopen.bmj.com/site/about/guidelines.xhtml

Biological specimens	33	Plans for collection, laboratory evaluation, and storage of biological specimens for genetic or molecular analysis in the current trial and for future use in ancillary studies, if applicable	____NA_______

*It is strongly recommended that this checklist be read in conjunction with the SPIRIT 2013 Explanation & Elaboration for important clarification on the items. Amendments to the protocol should be tracked and dated. The SPIRIT checklist is copyrighted by the SPIRIT Group under the Creative Commons "Attribution-NonCommercial-NoDerivs 3.0 Unported" license.

For peer review only

BMJ Open

Effectiveness and cost-effectiveness of a sustainable obesity prevention programme for pre-school children delivered at scale 'HENRY' (Health, Exercise, Nutrition for the Really Young): Protocol for the HENRY III Cluster Randomised Controlled Trial

Journal:	*BMJ Open*
Manuscript ID	bmjopen-2023-081861.R1
Article Type:	Protocol
Date Submitted by the Author:	13-Feb-2024
Complete List of Authors:	Bryant, Maria; University of York, Hull York Medical School; University of York, Health Sciences<br>Burton, Wendy; University of York, Department of Health Sciences<br>Collinson, Michelle; University of Leeds Clinical Trials Research Unit<br>Martin, Adam; Academic Unit of Health Economics, Leeds Institute of Health Sciences<br>Copsey, Bethan; University of Leeds Clinical Trials Research Unit,<br>Groves-Williams, Dawn; University of Leeds Clinical Trials Research Unit,<br>Foster, Alexis; University of Sheffield, School of Health and Related Research<br>Willis, Thomas A; University of Leeds<br>Garnett, Philip; University of York, School for Business and Society<br>O'Cathain, Alicia; University of Sheffield, School of Health and Related Research
**Primary Subject Heading**:	Public health
Secondary Subject Heading:	Nutrition and metabolism
Keywords:	Obesity, Community child health < PAEDIATRICS, Primary Prevention, PUBLIC HEALTH, Randomized Controlled Trial

SCHOLARONE™
Manuscripts

Effectiveness and cost-effectiveness of a sustainable obesity prevention programme for pre-school children delivered at scale 'HENRY' (Health, Exercise, Nutrition for the Really Young): Protocol for the HENRY III Cluster Randomised Controlled Trial

Professor Maria Bryant[1], Dr Wendy Burton[1], Ms Michelle Collinson[3], Dr Adam Martin[2], Dr Bethan Copsey[3], Dr Dawn Groves-Williams[3], Dr Alexis Foster[4]; Dr Thomas A Willis[3], Professor Philip Garnett[1], Professor Alicia O'Cathain[4]

[1] University of York, York, YO10 5DD

[2] Academic Unit of Health Economics, Leeds Institute of Health Sciences, University of Leeds, LS2 9JT

[3] Clinical Trials Research Unit, Leeds Institute for Clinical Trials Research, University of Leeds, LS2 9JT

[4] Sheffield centre for Health and Related Research (ScHaRR), University of Sheffield, S1 4DA

Corresponding author: Professor Maria Bryant, maria.bryant@york.ac.uk

Collaborator: Kim Roberts, HENRY, 6 Elm Place, Old Witney Road, Oxfordshire, OX29 4BD

Keywords: Obesity, prevention, protocol, community, childhood, systems

Word count: 5436

For peer review only

Abstract

Introduction

A fifth of children start school already overweight or living with obesity, with rates disproportionately impacting those living in the most deprived areas. Social, environmental and biological factors contribute to excess weight gain and programmes delivered in early years settings aim to support families to navigate these in order to prevent obesity. One of these programmes (HENRY) has been delivered in UK community venues (hereon named 'centres') in high deprivation areas since 2008 and aims to help families to provide a healthy start for their pre-school children. We aim to establish the effectiveness and cost-effectiveness of HENRY, including its potential role from a wider systems perspective.

Methods and analysis

This is a multi-centre, open-labelled, two group, prospective, cluster RCT, with cost-effectiveness analysis, systems-based process evaluation and internal pilot. Primary analysis will compare BMI z-score at 12 months in children (n=984) whose parents have attended HENRY to those who have not attended. Secondary outcomes include parent and staff BMI and waist circumference, parenting-efficacy, feeding, eating habits, quality of life, resource use and medium term (3 year) BMI z-scores (child and siblings). Eighty-two centres in ~14 local authority areas will be randomised (1:1) to receive HENRY or continue with standard practice. Intention-to-treat analysis will compare outcomes using mixed effects linear regression. Economic evaluation will estimate a within-trial calculation of cost-per unit change in BMI z-score and longer-term trajectories to determine lifelong cost savings (long term outcomes). A systems process evaluation will explore whether (and how) implementation of HENRY impacts (and is impacted by) the early years obesity system. An established parent advisory group will support delivery and dissemination.

Ethics and dissemination

Ethical approval has been granted by the University of York, Health Sciences' Research Governance Committee (HSRGC/2022/537/E). Dissemination includes policy reports, community resources, social media and academic outputs.

Registration details

ISRCTN16529380

Strengths and limitations of this study

- The HENRY III trial provides an opportunity to examine the effectiveness and cost-effectiveness of an intervention that has been publicly funded and delivered at scale for more than 10 years, including short, medium and long-term outcomes
- Our systems-based process evaluation will explore the role that community-based obesity prevention interventions play within the wider system, based on the understanding that factors that influence obesity are complex and multifaceted.
- The HENRY III trial has been designed following a successful feasibility study, in collaboration with local and national government partners, members of the public and a multi-disciplinary trial team.

For peer review only - http://bmjopen.bmj.com/site/about/guidelines.xhtml

- The trial's success is dependent upon recruitment of local authority areas, where additional funding will be required to commission the intervention.
- A mitigation strategy has been developed to reduce the risk of selection bias inherent in cluster designs. ,

For peer review only

For peer review only - http://bmjopen.bmj.com/site/about/guidelines.xhtml

Introduction

Reducing the prevalence of childhood obesity amongst people living in deprived areas is a public policy priority. Approximately one third of children (27.7% on average and up to 34.5% in higher deprivation areas) are defined as overweight (13.3%) or having obesity (14.4%). Health inequalities have also broadened, from a 6.3% difference in rates of overweight and obesity between the most and least deprived areas in 2019/20 to 10.7% in 2021/22 (1, 2).

Preventing excess weight in childhood is beneficial for health and wellbeing, as well as reducing medium- to long-term burden upon health services for those living with obesity, including mental health services and those used to treat and manage respiratory diseases such as asthma (3, 4). Obesity prevention during childhood can also reduce excess weight gain and obesity in later life (5, 6) which is difficult to reverse once established (7-9) and is a cause of numerous long-term, chronic health conditions. However, interventions to prevent obesity generally result in modest but inconsistent benefits (7-10). Given that obesity is caused by a wide range of physiological, psychological, environmental, economic and social factors, this is unsurprising and means that the role and cost-effectiveness of locally delivered programmes within a large and complex socio-economic and public health system is uncertain. Public Health England (PHE) advocated systems approaches; encouraging local areas to adopt a range of interconnecting interventions and policies inside and outside the healthcare sector to collectively tackle obesity and related health inequalities (11). It remains unknown what role individual interventions play in disrupting this system (12), and which of these interventions are most effective and cost-effective. Locally- delivered public health prevention programmes can be cost-effective (13); however, there is limited evidence specifically looking at obesity prevention delivered at scale. Further, while there has been an emergence of evidence that explores the role that understanding the obesity system has for adults (14-16) since the Foresight map was published (17), there is a lack of understanding of what an early years obesity system looks like and what the implications of understanding this might be.

One childhood obesity prevention programme which has been delivered at scale for many years in the UK is HENRY (Health, Exercise and Nutrition for the Really Young); a community-based programme, designed to alter early years settings, upskill the early years workforce and improve lifestyle behaviours of parents/carers (hereon called 'parents') and their preschool aged (under 5 years old) children. Although HENRY was designed to be a universal programme, it has been predominantly delivered in children's centres/community venues located in areas of high deprivation. Evidence suggests it has potential to impact on population obesity (18) but its effectiveness is not yet established. The current trial adopts a novel approach to evaluate the effectiveness and cost-effectiveness of the programme and explore the potential role of HENRY in disrupting the multiple factors that influence excess weight gain in the system. This novel approach places an emphasis on exploring if and how HENRY influences the system in which it operates (including system balancers which may reduce its potential for population impact).

Following on from a successful feasibility study (18) our current evaluation will consider child obesity outcomes in the short-term (using data collected in the trial), medium-term (using routinely collected data at 3 years) and longer-term (using economic modelling and secondary datasets).

Aims and objectives

For peer review only - http://bmjopen.bmj.com/site/about/guidelines.xhtml

Aim

To establish the effectiveness and cost-effectiveness of an obesity prevention programme delivered at scale, including its potential role from a wider systems perspective.

Primary objective of Randomised Controlled Trial (RCT)

1. The primary objective of the trial is to determine whether HENRY reduces child age- and sex - adjusted Body Mass Index (BMI) at 12 months.

Secondary objectives of RCT

1. To determine whether HENRY improves parent self-efficacy, eating behaviours, feeding behaviours, dental health, and quality of life
2. To explore whether HENRY influences rates of obesity in parents, siblings and staff (health practitioners)
3. To examine the social and physical environment in the children's centres
4. To monitor any safety issues from the intervention, including adverse events or unintended consequences

Internal pilot objectives of RCT

To assess centre recruitment, parent recruitment and HENRY programme delivery against pre-defined progression criteria.

Economic evaluation objectives

1. To explore the long-term effects on child BMI
2. To determine whether HENRY provides an overall cost saving (e.g. to the NHS)

Process evaluation objectives

1. To produce a map of the system within which HENRY operates and identify hypotheses about how this may be disrupted in response to HENRY
2. To analyse the system in which HENRY is embedded to understand how the system and its elements change over time in response to HENRY
3. To undertake a traditional process evaluation nested within the RCT to understand reach of HENRY within target population, potential contamination and how it has been implemented.

Methods and analysis

Trial design

A multi-centre, open-labelled, two group, prospective, cluster randomised controlled trial, with cost-effectiveness analysis, and embedded mixed methods complex systems evaluation and internal pilot (Figure 1). Eighty two eligible children's centres from within ~14 local authorities (depending on the number of centres per local authority) will be randomly allocated (1:1) to deliver HENRY or continue with standard practice (control). 984 eligible parents will be recruited.

[Figure 1 about here – separate file]

For peer review only - http://bmjopen.bmj.com/site/about/guidelines.xhtml

Setting and recruitment

Local authority and centre recruitment

We plan to recruit local authorities or other associations (e.g. NHS Trusts, private organisations) that are willing to commission HENRY across the UK, from which centres and parents will be recruited (Figure 2). In addition to inviting areas who actively express an interest in the intervention (independently from the trial) we will promote the trial in partnership with all NIHR Clinical Research Networks (CRN) in England and the equivalent organisations in the devolved nations. Eligible areas (criteria are detailed below) will then be asked to 1) nominate at least 2 (ideally 6 or more) centres which meet trial eligibility criteria, and 2) sign an agreement before entering the trial. We will record reasons for declining participation, and basic demographic information, from those authorities that choose not to participate.

Local authorities will be asked to nominate approximately twice the number of centres for which they wish to commission HENRY, so that approximately half can be randomised to receive HENRY and half can be randomised to control. Although there are no exclusions based on the demographics of centres, location will be monitored and commissioners encouraged to nominate centres to include a range of diverse social and environmental characteristics. Centre Managers will be given information about the research and asked to provide consent for their centre to participate through a collaboration agreement.

[Figure 2 about here]

Parent/carer and staff recruitment

Centre staff will invite all parents who are booked to attend a HENRY programme to take part in the research (and will be renumerated per participant based on NIHR CRN service support costs (19)). If interested, they will be screened for eligibility and will be asked to provide informed consent to take part. Those who decline will still be able to attend the HENRY intervention if they wish. Screening and recruitment will aim to start at least 6 weeks prior to the start of each HENRY programme. Programmes typically enrol an average of 8 parents, of whom our design aims to recruit an average of 4 parents. Once consented, blinded data collection staff will contact participants to arrange the baseline visit and complete trial registration. A similar process will recruit parents from the control centres. Here, parents who attend other programmes (e.g. stay and play sessions) will be invited to take part within the same timeframes. Parents will also have the opportunity to self-refer into the trial via recruitment posters displayed in the centres. A £30 shopping voucher will be offered to participants in recognition of their contribution (£15 at baseline, £15 at follow-up). Parent consent will include five options: consent to take part in the trial only, consent to provide height and weight measurements, consent to provide HENRY programme attendance data (if attending a HENRY programme), consent to be contacted about taking part in a process evaluation interview, and consent to share routinely collected National Child Measurement Programme (NCMP) data after 3 years for the purposes of the economic evaluation.

Staff recruitment will occur once they have completed site training. Those agreeing to take part will provide their height, weight and waist circumference, which they can self-measure. Staff will also have the opportunity to consent to be contacted about taking part in a process evaluation interview.

Eligibility criteria

For peer review only - http://bmjopen.bmj.com/site/about/guidelines.xhtml

Local authorities can be new to HENRY or already commissioning HENRY, provided they have at least 2 centres (ideally 6) meeting centre eligibility criteria. Local authorities using external teams outside of the centre to deliver HENRY programmes (e.g. health visitors) will be eligible, in addition to those wishing to train internal centre staff to deliver programmes (the most common model). Local authorities without coverage of a National Institute of Health Research (NIHR) local clinical research network (LCRN; the teams responsible for collecting trial data from parents and staff) will not be eligible.

Centres: Any type of centre or other early years setting such as a nursery or community venue will be eligible to take part provided that they do not already deliver HENRY, and their staff have not received HENRY training (within the past two years). This includes both public and private nurseries. Centres where staff are shared between nominated centres will not be eligible. Centres must aim to run HENRY programmes starting within 12 weeks of training completion and plan to run 3 programmes during their trial participation period (approximately 18 months). They should be in geographically separate areas to protect against contamination (judged on a case by case basis) and managers must agree to support participant recruitment within their centres. If the centre operates as part of a cluster, that cluster must be deemed to be HENRY naive. HENRY naive clusters are defined as: a group of centres within a cluster that do not include any centres that are (a) currently delivering HENRY or (b) have been trained to, or delivered HENRY within the past 2 years.

Centres that are either currently delivering HENRY or have previously delivered HENRY in the last two years are not eligible.

Parents/carers: The target population for the intervention are parents/carers of preschool children. Parents may not be registered more than once but they may be screened on more than one occasion if not registered following first screening, as both eligibility and willingness to participate may change. Parents must have at least 1 child aged 6 months - 5 years (18 months - 6 years at 12 month follow-up) at the time of starting the programme. If more than one child in a family fulfils eligibility criteria, the youngest child (by birth timing if twins) will be considered as the reference child (from which data will be collected). Parents must be willing to attend the programme sessions (intervention centres) and willing to provide data in accordance with the data collection protocol. Parents will be provided with full details of the data collection requirements in advance so that they can make informed decisions as to whether to participate. They must either speak English, or bring their own interpreter with them (e.g. family member) each time they need to respond to questionnaires for trial purposes. Where available, non-English speaking parents will also be able to use local interpreters (provided by the centre or through the local clinical research network). Parents with severe learning difficulties that preclude them taking part in group sessions in which they need to be able to read and write will not be eligible. Those whose reference child is tube fed or with other known clinical conditions likely to affect growth over the period of the trial (e.g. coeliac disease) and those who have attended a HENRY group for a previous child will also be ineligible.

We have developed a recruitment strategy based on learning from our feasibility study (18) which includes screening and consenting by centre staff (who are familiar to families) and detailed training for centre staff, including clear timelines and expectations. For example, in order to meet targets, a minimum of four parents should be recruited within a six week window prior to the start of each HENRY programme (and at an equivalent time in control centres).

For peer review only - http://bmjopen.bmj.com/site/about/guidelines.xhtml

All centre staff will be invited to participate in the research including those directly and indirectly involved in delivering HENRY programmes.

Randomisation and blinding

Following fully signed local authority (and service provider if applicable) and centre agreements, participating centres within each local authority will be randomised to HENRY or control in a 1:1 allocation ratio by the Leeds Clinical Trials Research Unit (CTRU). Minimisation, incorporating a random element, will be used to ensure the treatment groups are well balanced for the following characteristics:

- Size of centre (≤8 / >8 permanent centre members of staff, not including staff using the centre such as Health Visitors, nursery workers etc.,);
- Area level ethnicity (<80% / ≥80% White British using Census data based on centre postcode) and;
- Area level deprivation (≤10% / >10% ranking within Index of Multiple Deprivation at the Lower Layer Super Output Area).

Parents and centre staff will not be blinded due to the nature of the intervention. Data collection staff will be blinded to centre allocation (and thus the participants' treatment arm).

Active intervention

HENRY was set up with Department of Health funding in 2008 and has been widely commissioned by more than 50 areas (training >15,000 practitioners and providing programmes to ~24,500 parents). It includes core practitioner training and group facilitation training (www.henry.org.uk)(20).

HENRY is delivered primarily within Sure Start centres and other children's centres, as well as other community settings, including schools, mosques and churches. Local authority areas can choose to have HENRY programmes delivered directly by HENRY central teams (less common approach), pay to commission training, licensing and support from HENRY (most common approach), offer a blended approach, or provide training to local staff to deliver programmes (most common in larger local authority areas).

Intervention training for practitioners:

Core practitioner training supports staff to deliver the HENRY approach, which incorporates evidence-based behaviour change models, including the Family Partnership model, motivational interviewing and solution focused support, with information about a healthy start that is consistent with national guidance. This training is designed to allow staff to integrate evidence based models to develop motivation and support lifestyle change for families. This can then be embedded into all interactions with families, in addition to supporting positive changes to the centre environment (space to play, freely available water, food policies etc.). Training is provided to health visitors, dieticians and staff (e.g. at children's centres, community centres/hubs - hereon called 'centres') allowing parental support to be an intrinsic part of their role, whilst influencing culture and policy within early years settings. Training is designed to consistently influence the environment and practice immediately following training.

Facilitator training is delivered to a (usually) smaller selection of staff who have attended core training to certify them to deliver small group sessions 'Right from the Start'. This training enables the delivery of an 8-week universal 'Right from the Start' programme to parents to provide practical skills in authoritative parenting skills, increasing self-esteem, adopting healthy family lifestyles, goal setting, oral health, active play, portion sizes, and learning about food labels.

For peer review only - http://bmjopen.bmj.com/site/about/guidelines.xhtml

Training can be delivered in-person or remotely, depending on commissioner and facilitator preference. To allow a richer level of communication, the in-person training can be completed in a shorter time frame (2-day in-person training is delivered over 6 weeks remotely).

Programme delivery to parents:

The 'Right from the Start' programme is delivered to groups of approximately 8-10 parents over 8 sessions. Each interactive session focuses on a separate theme and includes resources for families to take home. Each centre delivers 2-3 'Right from the Start' group programmes per year, each consisting of 8 x 2-hour sessions.

Programmes can be delivered remotely or face-to-face. We will include both delivery modes in the proposed research. This will allow us to be flexible depending on external issues, in addition to supporting an evaluation of a pragmatic intervention. Our analysis plan includes consideration of different delivery approaches.

Parents with a child aged up to 5 years are eligible to attend HENRY. The programme can be tailored to specific groups or parents, for example, to align with dietary practices for different ethnicities or religion. Language needs are addressed locally depending on the population needs, with some areas providing dedicated support. Without this, the other approach is to invite parents to attend with a friend or family member to support translation and other activities. Although considered to be a Universal programme, practitioners often refer families 'at risk' to HENRY and the delivery model within children's centres allows parents from the most deprived neighbourhoods to attend.

Parents receive a standardised manual as part of the programme. To monitor adherence, session attendance will be recorded for parents who consent.

Control intervention

Staff within control centres will not receive HENRY training and will continue to deliver usual programmes or 'standard practice' (e.g., 'stay and play', 'cook and eat'). In short, this means that families registered to take part in the research will receive the standard level of support provided within their community / centres. Services are aimed at supporting families with a focus on the most disadvantaged families. These vary between and within local areas but usually include access to health visiting teams, breastfeeding support, parenting advice and access to specialist services including speech and language therapy.

Outcomes

The primary outcome for the trial is child age and sex adjusted BMI z-score) assessed at 12 months post parent registration.

Secondary outcomes will be assessed at the same timepoint (defined short-term), after 3 years (medium-term) and will be forecasted across the life-course (longer-term). Individual level outcomes are summarised in Table 1 (See Supplementary Table 1 for a full list of outcomes and measures)

For peer review only - http://bmjopen.bmj.com/site/about/guidelines.xhtml

Table 1 Outcome data collection summary (see Supplementary Table 1 for a full list of data and outcomes)

Outcomes	Measures	Collected by	Participant / staff Baseline	Short term follow-up (12 months post registration)	Medium-term follow-up (3 years)[a]	Longer term follow-up[b]
Child age and sex adjusted BMI Z-score, child height, child weight, unadjusted BMI and weight/BMI percentiles	Measured	LCRN[d] (baseline & 12 M follow-up)<br><br>Longer term: CTRU[c] accessing routine data	x	x	x	
Sibling age and sex adjusted BMI, height (m), weight (kg), unadjusted BMI and weight/BMI percentiles		CTRU accessing routine data			x	
Parent self-efficacy	Dumka (27)	LCRN	x	x		
Family eating / activities	Golan (28)	LCRN	x	x		
Feeding questionnaire	Baughcum (29)	LCRN	x	x		
Dental health (child)	Dental questionnaire	LCRN	x	x		
Parent height & weight:	Measured	LCRN	x	x		
Parent waist circumference	Measured	LCRN	x	x		

For peer review only

Outcomes	Measures	Collected by	Participant / staff Baseline	Short term follow-up (12 months post registration)	Medium-term follow-up (3 years)[a]	Longer term follow-up[b]
**Staff:**						
Staff screening	Staff screening form	Staff self complete at centre				
Staff height & weight	Measured	Self-measure	x	x		
Staff waist circumference	Measured	Self-measure	X	x		
**Routine data:**						
NCMP Child and sibling data (trial participants)		CTRU			x	
NCMP regional child data (not trial participants)		CTRU			x	

[a] Medium term outcomes will gathered from routinely collected data (from health visitors and/or the National Child Measurement Programme)

[b] Longer term outcomes will be based on matched cohorts of (Millennium Cohort Study (MCS ((21)).

[c] Clinical Trials Research Unit

[d] Local Clinical Research Network (England only). Other devolved nations will train researchers/equivalent staff to collect data)

For peer review only - http://bmjopen.bmj.com/site/about/guidelines.xhtml

Data collection

Table 1 provides information about the types of data collected and how these are collected. Baseline and follow-up data (12 months) will be collected within participant homes by trained Local Clinical Research Network (LCRN) staff (or equivalent trained staff in the devolved nations). Where preferred by the participant, there will be options to collect data in the children's centre. The majority of questionnaires completed during home visits will be interviewer administered. Centre level outcomes will be completed by a nominated member of centre staff (usually the centre manager or HENRY facilitator). Staff level data will be self-completed and entered directly on to the online database.

Sample size

41 centres per arm (from 10-14 local authorities), each recruiting 12 parents on average (4 parents from 3 programmes, 984 parents in total) will provide 90% power to detect a small standardised effect size of 0.27 as per previous trials [25-28] for BMI z-score at a 5% significance level, assuming an ICC of 0.03 [16, 25, 29-31] to account for clustering by centre, a coefficient of variation of 0.48 to account for variation in centre recruitment and 20% loss to follow-up (18).

Withdrawal of consent

Centres and/or local authorities can withdraw at any point during the trial. Data collected up to the time of withdrawal will be retained for analysis and data from parents and staff will still be collected provided they have not withdrawn consent themselves. Centres may stop delivering the HENRY programme during the trial period independently from the trial (e.g. centre closures, restructuring). Trial procedures will continue in this eventuality and all recruited parents and staff will remain in the trial (and data will continue to be collected from them) unless they actively withdraw. Where parents or staff wish to withdraw, there will be clarification whether this is withdrawal from short term trial data collection, or from medium term trial data collection (using routine data) or a combination of these. Non-attendance at HENRY intervention sessions are not classed as a withdrawal from the trial. All parents who withdraw from HENRY intervention or who do not attend the intervention will still be followed–up for data collection unless they specifically express a wish to withdraw from trial processes.

Data analysis plan

Analysis will be carried out on the intention-to-treat (ITT) population defined as all local authorities / centres randomised and all parents registered to the trial, regardless of adherence to the protocol, withdrawal of consent or losses to follow-up. A two-sided 5% significance level will be used for statistical endpoint comparisons. No interim analyses are planned, except for safety data that are required for review by the Data Monitoring and Ethics Committee.

The flow of local authorities, centres and participants through the trial will be presented in a Consolidated Standards of Reporting Trials diagram.

The primary outcome, child age and sex adjusted BMI (BMI z-score), will be analysed using a multi-level linear regression with children nested within centres, and centres treated as a random effect. The model will be adjusted for the following fixed effects: centre-level stratification factors, important parent-level and child-level covariates (e.g. baseline child BMI z-score and sex, parent BMI), and other relevant known predictors of outcome. Missing data will be imputed at the individual participant level where appropriate. Estimated mean differences will be reported with

For peer review only - http://bmjopen.bmj.com/site/about/guidelines.xhtml

95% confidence intervals, p-values and ICCs. Model diagnostics will be used to check the underlying assumptions of the model and alternative methodology will be used if required.

Sensitivity analyses of the primary endpoint will be conducted to assess the impact of missing data, the choice of imputation model and the missing at random assumption, as appropriate. If contamination between intervention and control centres is identified, a sensitivity analysis excluding the relevant control centres will be conducted.

If numbers allow, exploratory subgroup analysis will examine differences in intervention effect between different socio-economic and ethnic groups, and also between online and face-to-face delivery of HENRY.

For secondary outcomes, summary statistics will be presented at baseline and 12 months post-parent registration overall and by arm (means, standard deviations, medians, minimum, maximum, and quartiles for continuous variables, and counts and percentages for categorical variables).

Medium-term analysis (3-years post parent recruitment) will compare regional population level BMI z-score (trial local authority areas) with that of trial participants and siblings (HENRY and control) to investigate differences in BMI z-score. Analysis will use the same approach as the primary outcome for different outcome types, using multi-level regression with multiple imputation for missing data.

Internal pilot

Descriptive analysis of the internal pilot against progression criteria will take place at three separate time points as described in Table 2 and discussed with the trial steering committee to inform a decision on the modification or continuation of the trial.

Table 2. Internal pilot progression criteria

Criteria	Green (go)	Amber (review)	Red (stop)
**Centre Recruitment**<br>Centres open within 12 months of starting centre recruitment<br>(including data up to the end of month 19). A centre will be defined as open if it has been randomised and is open to recruitment.	≥54	42-53	<42
**Parent Recruitment**<br>Average number of parents recruited per programme / equivalent<br>(including data up to the end of month 23, allowing for 6 months of parent recruitment).	≥4	3 to <4	<3
**HENRY Programme delivery**<br>Percentage of intervention centres having started delivery of at least 1 programme<br>(including data up to the end of month 27, allowing for 18 months from starting centre recruitment). The denominator will include all centres allocated to the intervention arm. The numerator will be all of those intervention centres who have started to deliver at least one programme,	≥80%	50% to 80%	<50%

For peer review only - http://bmjopen.bmj.com/site/about/guidelines.xhtml

Criteria	Green (go)	Amber (review)	Red (stop)
defined as delivering at least one session to parents.			

Economic evaluation

Economic analysis will be conducted in three stages: (1) 12 month short-term within-trial cost-effectiveness analysis of the incremental cost per unit change in BMI z-score; (2) Three year cost-effectiveness analysis of the incremental cost per unit change in BMI z-score with additional routinely collected National Child Measurement Programme data; and (3) Longer-term estimates of BMI z-score trajectories and healthcare use using a matched cohort of Millennium Cohort Study (MCS) participants (21). The MCS dataset provides a unique and high-quality resource that enables us to project long-term BMI trajectories and healthcare resource use into early adulthood at the individual-level.

The overarching aim of the economic analysis is to reduce decision uncertainty about whether or not HENRY should be commissioned. As we increase the time horizon of the analysis, it is anticipated that decision uncertainty will be reduced in the sense that it will become much clearer whether or not HENRY should be commissioned. Primary analyses will adopt an NHS and local authority perspective. Supplementary analyses will adopt a wider societal perspective by assessing household costs and productivity losses. Spill over benefits to parents will also be captured by calculating differences between the two treatment groups in terms of quality adjusted life years (QALYs) over 12 months. Utility scores will be measured using both the EQ-5D-5L (22) and the ICECAP-A (23). All analyses will be conducted using the ITT population. Seemingly unrelated regression will be used to account for the correlation between costs and outcome measures. Multilevel models will be used to account for children nested within centres. Decisions about which child-level, parent-level and centre-level covariates to include in the models will be made after assessing differences in baseline characteristics, and through discussion with the trial statisticians. Patterns (and reasons) of missing data will be investigated in collaboration with the trial statisticians and appropriate imputation techniques will be used. Supplementary analyses will assess whether there are differences in costs and effectiveness by the intervention delivery method by including an interaction term between method (i.e., online vs. face-to-face) and the treatment variable. In order to address whether or not the intervention is equally cost-effective among children living in the most deprived areas and households, analyses will also use interaction terms for household-level socioeconomic status and for area level deprivation (≤ 10%/> 10% ranking within Index of Multiple Deprivation at the Lower Super Output Area). To assess whether a parent/carer's propensity to commit to engage with HENRY was affected by their attitude to risk or perceptions about their long-term health prospects, analyses will also use interaction terms for self-reported measures of these factors based on questions used in the English Longitudinal Study of Ageing (ELSA) (24) and German Socioeconomic Panel (SOEP) (25).

Uncertainty in our cost-effectiveness estimates will be characterised by presenting bootstrapped estimates on CEACs using a wide range of different cost-effectiveness thresholds (£/BMI change). Whilst acknowledging that the true WTP threshold is unobserved, the choice of thresholds would be based on existing literature on the cost-effectiveness of comparable interventions and expert opinion. A version of the CEAC which shows the probability of the intervention being cost-saving (i.e. in the SE quadrant), regardless of the willingness to pay (WTP) threshold, will also be presented. One way sensitivity analyses will include an assessment of varying the intervention cost and of using alternative methods and approaches to matching the trial data with participants in the Millennium

For peer review only - http://bmjopen.bmj.com/site/about/guidelines.xhtml

Cohort Study (MCS) dataset. Results for all economic analyses will be reported in accordance with the Consolidated Health Economic Evaluation Reporting Standards (CHEERS) guidelines (26) using Stata (StataCorp LLC Version 18.0) and/or R (R Version 4.3).

Systems-based process evaluation

HENRY is a complex intervention that can be viewed as an event within a system (27). Therefore, we will adopt a complex systems perspective, using a framework for qualitative systems process evaluations (28) and quantitative systems evaluation, embedded within a mixed methods process evaluation (29). The evaluation will consist of the two-stage qualitative systems approach: (1) initial systems mapping, and (2) analysis of the system within which HENRY is embedded. It will also include a traditional process evaluation to understand context, mechanisms and implementation of HENRY (29) and quantitative systems modelling.

The systems map will be constructed using data collected at a stakeholder workshop (n=30-40) at the start of the RCT in which we will identify: structure (e.g. levels of national, regional and local); elements (e.g. national public health priorities around childhood health, obesity and parenting; local authority priorities and funding situation; health visitor responsibilities; organisations providing HENRY; welfare benefits systems; historical events affecting childhood obesity); relationships and interactions between elements; and boundaries (what is inside and outside the system). Invited stakeholders will include (but will not be restricted to) childcare practitioners, public health specialists, commissioners, parents, health visitors and local and national early years organisations/delivery teams. The overall map may ultimately consist of a number of sub-maps that will highlight causal inter-relationships between each of the elements of these areas. It may also present HENRY at different levels (the local authority, centre, parent/child and other) identified via this research. These maps will comprise fundamental systems thinking 'building blocks' in the form of causal-loop diagrams and/or stock-and-flow structures as appropriate. Each set of diagrams (or system maps) will seek to relate parameter/variable components of the local system through positive/negative causality and ordinalities such that a dynamic representation of inter-relationships can be viewed graphically.

The second stage of qualitative systems process evaluation will explore how the system and its elements change over time in response to HENRY. Six months prior to the end of the RCT, we will undertake qualitative telephone interviews/virtual interviews with 20-24 national, regional and local key stakeholders representing elements of the Stage 1 map to discuss the system structure and elements, how they have changed over time, the way the system responded to HENRY, and how responses amplified or dampened HENRY's impacts. We will also explore how HENRY affected or was affected by potential strategies for addressing health inequalities.

To complement McGill's qualitative systems process evaluation, we will also focus on understanding the reach of HENRY within the target population, how HENRY works, potential contamination, and how it has been implemented within the RCT (29). We will undertake semi-structured interviews with staff providing HENRY (n=12-16) to explore their views of the feasibility of HENRY, how it has been delivered in different centres over time (variation in implementation), access to HENRY for different socio-economic and ethnic minority groups (reach), perceptions of differential health outcomes for different socio-economic and ethnic minority groups (that is, how it addresses health inequalities or not), and views of potential contamination in the RCT. We will also undertake semi-structured interviews with parents (n=20-24) attending HENRY to explore acceptability of the intervention and perceptions of how it facilitated health improvement or not. In both sets of interviews, we will explore mechanisms of impact (how HENRY has affected parenting, nutrition and weight of children), facilitators and barriers to delivering or attending HENRY, reach in terms of

For peer review only - http://bmjopen.bmj.com/site/about/guidelines.xhtml

accessing those most in need, and implementation in practice. We will also include a systems lens by putting context at the centre of the interviews and explore the impact of system elements on HENRY, specifically asking about how elements of the system documented in Stage 1 interacted with HENRY. These interviews may affect our understanding of why HENRY was implemented in different ways in different localities or at different times, and the extent to which health inequalities have been addressed by HENRY.

Quantitative process data will be gathered to summarise attendance and drop out by centre and overall and will support the monitoring and evaluation of contamination. Thus, in addition to exploring contamination through qualitative interviews, data on staff movement and sharing of HENRY related messages will be captured. Key to this will be an online HENRY-trained staff survey at two time periods (towards the end of the internal pilot and at the end of the RCT intervention period) asking whether, how and when staff shared HENRY practice outside their centre.

Qualitative data will be analysed prior to the trial outcome analysis using the Framework approach. An initial thematic framework will be based on familiarisation of a range of transcripts, analysis of qualitative interviews from the feasibility study (30), and the process evaluation framework (mechanisms, context and implementation) (29). Quantitative evaluation will combine trial and process data to apply a causality-based model of obesity prevention to develop a systems archetype (31). Analysis will incorporate Morphological Analysis and Fuzzy Cognitive Mapping techniques (32) to evaluate and assign inter-relationships between components through causal weights and network directionality. We will examine the range of operating conditions of the HENRY system model to classify its overall dynamic behaviour (identifying which Systems Archetype HENRY most closely resembles (31); providing a basis for comparison to other obesity models (e.g., Foresight) to explain how HENRY may disrupt obesity prevalence in childhood).

Patient and Public involvement

We will consult closely with a diverse group of parents with young children from a local children's centre who supported the design and research processes for our funding application. Activities will include workshop-style meetings timed to project milestones and in line with trial steering committee meetings (approximately twice per year of the trial). Each workshop will have a specific aim and associated set of activities including brainstorming and group discussion. We will share workshop outcomes with research team members, including the steering committee, and, where applicable, incorporate them in research activities. To begin, the group will be asked to consider their needs for involvement, including training or practical requirements. The dedicated public involvement lead (WB) will be responsible for setting and overseeing the overall strategy. Proposed activities will be embedded in all stages of the research. For example, during recruitment, we will discuss unanticipated barriers (e.g., Covid related issues) so that we can help mitigate these. The group will provide input into the process evaluation work; for example, ensuring concepts and language used during systems mapping workshops is appropriate. They will also be involved in interpreting trial results in a way most relevant to parents and help to design and disseminate trial outputs. Parents will be renumerated for their time using NIHR guidelines (33).

Ethics and dissemination

The trial will adhere to ethical principles, approach, aims and methods of the ESRC research framework. Ethical approval was obtained by the University of York, Health Sciences' Research Governance Committee (HSRGC/2022/537/E). HRA approval was also obtained to allow NHS (e.g. children's centres sitting within integrated care organisations) sites to take part in the trial (Integrated Research Application System (ID:317992). Serious adverse events are not anticipated;

For peer review only - http://bmjopen.bmj.com/site/about/guidelines.xhtml

however, we have convened an independent trial steering committee (TSC) and separate data monitoring committee (DMEC) to monitor trial progress and adherence (TSC) and ethical issues and safety (DMEC).

Our dissemination strategy includes both the production of regular progress reports and outputs to share with local and national governments, and final reports and publications. For example, we will disseminate findings of the systems work via meetings and conferences, and via publications (i.e., not restricted to the end of trial). Our strategy also includes regular public facing newsletters, social media outputs and attendance at festival events. Final outputs will be shared via a dissemination event, policy briefings and academic publications/presentations.

Author contributions

MB: Conceptualisation, Funding acquisition, Oversight (study lead), Methodology, Writing- Original draft, Writing- Reviewing and Editing; **WB**: Conceptualisation, Funding acquisition, project management, PPI lead, Writing- Original draft, Writing- Reviewing and Editing; **MC**: Funding acquisition, Methodology (statistical lead), Writing- Original draft, Writing- Reviewing and Editing; **AM**: Funding acquisition, Methodology (economics lead), Writing- Original draft, Writing- Reviewing and Editing; **BC**: Funding acquisition, Methodology (analysis), Writing- Original draft, Writing- Reviewing and Editing; **DGW:** Methodology (Trial management), Writing- Original draft, Writing- Reviewing and Editing; **AF**: Methodology (systems process evaluation), Writing- Original draft, Writing- Reviewing and Editing; **TW**: Methodology (Trial operations), Writing- Original draft, Writing- Reviewing and Editing; **PG**: Methodology (systems quantitative evaluation lead), Writing- Reviewing and Editing; **AOC:** Funding acquisition, Methodology (systems process evaluation lead), Writing- Original draft, Writing- Reviewing and Editing;

Competing interests

No authors report any conflicts of interest

Acknowledgements

Funding

This study/project is funded by the NIHR [name of NIHR programme (NIHR135081]. The views expressed are those of the authors and not necessarily those of the NIHR or the Department of Health and Social Care.

Patient and Public involvement

We acknowledge our parent advisory group for their support in developing the intervention and ongoing advice in study design and recruitment, including Lauren Surtees, Sarah Green, Abeer Mukhta and Lina Alrefaai.

Trial Steering Committee (TSC) /Data Monitoring Committee (DMEC)

We thank the members of the TSC, including Professor Peymane Adab (TSC Chair, University of Birmingham), Professor Amelia Lake (Teeside University), Professor Laura Cornelsen (London School of Hygiene and Tropical Medicine), Dr. Nasima Akhter (Teeside University) and Lina Alrefaai (parent representative), Paul Atkin (CRN Yorkshire), Professor Simon Coulton (University of Kent), Lauren Surtees (parent representative). We are also grateful to our DMEC members, Dr Sarah Seaton

For peer review only - http://bmjopen.bmj.com/site/about/guidelines.xhtml

(University of Leicester), Professor Miranda Pallan (University of Birmingham) and Dr Sarah Crozier (University of Southampton).

References

1. NHS Digital. The National Child Measurement Programme England 2020/21 School Year 2021 https://digital.nhs.uk/data-and-information/publications/statistical/national-child-measurement-programme/2020-21-school-year. Accessed 21.11.22
2. Browne NT, Snethen JA, Greenberg CS, Frenn M, Kilanowski JF, Gance-Cleveland B, et al. When Pandemics Collide: The Impact of COVID-19 on Childhood Obesity. J Pediatr Nurs. 2021;56:90-8.
3. Hasan T, Ainscough TS, West J, Fraser LK. Healthcare utilisation in overweight and obese children: a systematic review and meta-analysis. BMJ open. 2020;10(10):e035676.
4. Sharma V, Coleman S, Nixon J, Sharples L, Shield J, Rutter H, et al. Systematic Review and Meta-Analysis of Co-morbidities Associated with Child and Adolescent Obesity (aged 5-18). Obesity Reviews. 2019;20:1341-9.
5. Pandita A, Sharma D, Pandita D, Pawar S, Tariq M, Kaul A. Childhood obesity: prevention is better than cure. Diabetes Metab Syndr Obes. 2016;9:83-9.
6. Public Health England. PHE Strategy 2020-25. 2019 Sept 2019. https://assets.publishing.service.gov.uk/government/uploads/system/uploads/attachment_data/file/830105/PHE_Strategy__2020-25__Executive_Summary.pdf Accessed
7. Redsell S, Atkinson P, Nathan D, Siriwardena N, Swift J, Glazebrook C. Primary prevention of childhood obesity: views from primary care. Archives of Disease in Childhood. 2011;96(Suppl 1):A9-A11.
8. Redsell SA, Edmonds B, Swift JA, Siriwardena AN, Weng S, Nathan D, et al. Systematic review of randomised controlled trials of interventions that aim to reduce the risk, either directly or indirectly, of overweight and obesity in infancy and early childhood. Maternal & child nutrition. 2016;12(1):24-38.
9. Hillier F, Pedley C, Summerbell C. Evidence base for primary prevention of obesity in children and adolescents. Bundesgesundheitsblatt, Gesundheitsforschung, Gesundheitsschutz. 2011;54(3):259-64.
10. Brown T, Moore THM, Hooper L, Gao Y, Zayegh A, Ijaz S, et al. Interventions for preventing obesity in children. Cochrane Database of Systematic Reviews. 2019(7).
11. Public Health England. Whole systems approach to obesity: a guide to support local approaches to promoting a healthy weight. 2019 July. https://www.gov.uk/government/publications/whole-systems-approach-to-obesity Accessed 21.11.22
12. Carey G, Malbon E, Carey N, Joyce A, Crammond B, Carey A. Systems science and systems thinking for public health: a systematic review of the field. BMJ open. 2015;5(12):e009002.
13. Owen L, Morgan A, Fischer A, Ellis S, Hoy A, Kelly MP. The cost-effectiveness of public health interventions. Journal of Public Health. 2011;34(1):37-45.
14. Finegood DT, Merth TDN, Rutter H. Implications of the Foresight Obesity System Map for Solutions to Childhood Obesity. 2010;18(S1):S13-S6.
15. Keaver L, Webber L, Dee A, Shiely F, Marsh T, Balanda K, et al. Application of the UK foresight obesity model in Ireland: the health and economic consequences of projected obesity trends in Ireland. PLoS One. 2013;8(11):e79827.
16. Morris MA, Wilkins E, Timmins KA, Bryant M, Birkin M, Griffiths C. Can big data solve a big problem? Reporting the obesity data landscape in line with the Foresight obesity system map. International Journal of Obesity. 2018.

For peer review only - http://bmjopen.bmj.com/site/about/guidelines.xhtml

17. McPherson K, Marsh T, Brown M. Foresight report on obesity. Lancet. 2007;370(9601):1755; author reply
18. Bryant M, Burton W, Collinson M, Farrin A, Nixon J, Stevens J, et al. A cluster RCT and process evaluation of an implementation optimisation intervention to promote parental engagement enrolment and attendance in a childhood obesity prevention programme: results of the Optimising Family Engagement in HENRY (OFTEN) trial. Trials. 2021;22(1):773.
19. NIHR. NIHR (National Institute for Care and Research) CRN Recruitment Policy Document. 2023. https://www.nihr.ac.uk/documents/nihr-crn-recruitment-policy-document/11347 Accessed
20. Willis TA, Potrata B, Hunt C, Rudolf MC. Training community practitioners to work more effectively with parents to prevent childhood obesity: the impact of HENRY upon Children's Centres and their staff. Journal of human nutrition and dietetics : the official journal of the British Dietetic Association. 2012;25(5):460-8.
21. Connelly R, Platt L. Cohort Profile: UK Millennium Cohort Study (MCS). International Journal of Epidemiology. 2014;43(6):1719-25.
22. Devlin NJ, Shah KK, Feng Y, Mulhern B, van Hout B. Valuing health-related quality of life: An EQ-5D-5L value set for England. Health economics. 2018;27(1):7-22.
23. Al-Janabi H, N Flynn T, Coast J. Development of a self-report measure of capability wellbeing for adults: the ICECAP-A. Quality of Life Research. 2012;21(1):167-76.
24. Minicuci N, Naidoo N, Corso B, Rocco I, Chatterji S, Kowal P. Data Resource Profile: Cross-national and cross-study sociodemographic and health-related harmonized domains from SAGE plus CHARLS, ELSA, HRS, LASI and SHARE (SAGE+ Wave 2). Int J Epidemiol. 2019;48(1):14-j.
25. Wagner GG, Frick JR, Schupp J, Berlin J. The German Socio-Economic Panel Study (SOEP): Scope, Evolution and Enhancements, SOEPpapers on Multidisciplinary Panel Data Research, No. 1. Deutsches Institut für Wirtschaftsforschung (DIW)2007.
26. Husereau D, Drummond M, Petrou S, Carswell C, Moher D, Greenberg D, et al. Consolidated Health Economic Evaluation Reporting Standards (CHEERS) statement. BMJ : British Medical Journal. 2013;346:f1049.
27. Hawe P, Shiell A, Riley T. Theorising interventions as events in systems. Am J Community Psychol. 2009;43(3-4):267-76.
28. McGill E, Marks D, Er V, Penney T, Petticrew M, Egan M. Qualitative process evaluation from a complex systems perspective: A systematic review and framework for public health evaluators. PLOS Medicine. 2020;17(11):e1003368.
29. Moore G, Audrey S, Barker M, Bond L, Bonell C, Hardeman W, et al. Process evaluation of complex interventions: Medical Research Council guidance. BMJ. 2015;350(h1258).
30. Ritchie J, Spencer L. The Qualitative Researcher's Companion. 2002 2023/10/27. Thousand Oaks

Thousand Oaks, California: SAGE Publications, Inc. Available from: https://methods.sagepub.com/book/the-qualitative-researchers-companion.
31. Braun W. The System Archetypes: The Systems Modeling Workbook2002 16.07.21.
32. Sharif A., Irani Z. Fuzzy Cognitive Mapping for MIS Decision-Making. . In: (eds) KC, editor. Fuzzy Applications in Industrial Engineering. 201. Berlin, Heidelberg: Springer; 2006.
33. NIHR. Payment guidance for researchers and professionals. 2023. https://www.nihr.ac.uk/documents/payment-guidance-for-researchers-and-professionals/27392 Accessed

For peer review only - http://bmjopen.bmj.com/site/about/guidelines.xhtml

FIGURE 1: TRIAL SUMMARY

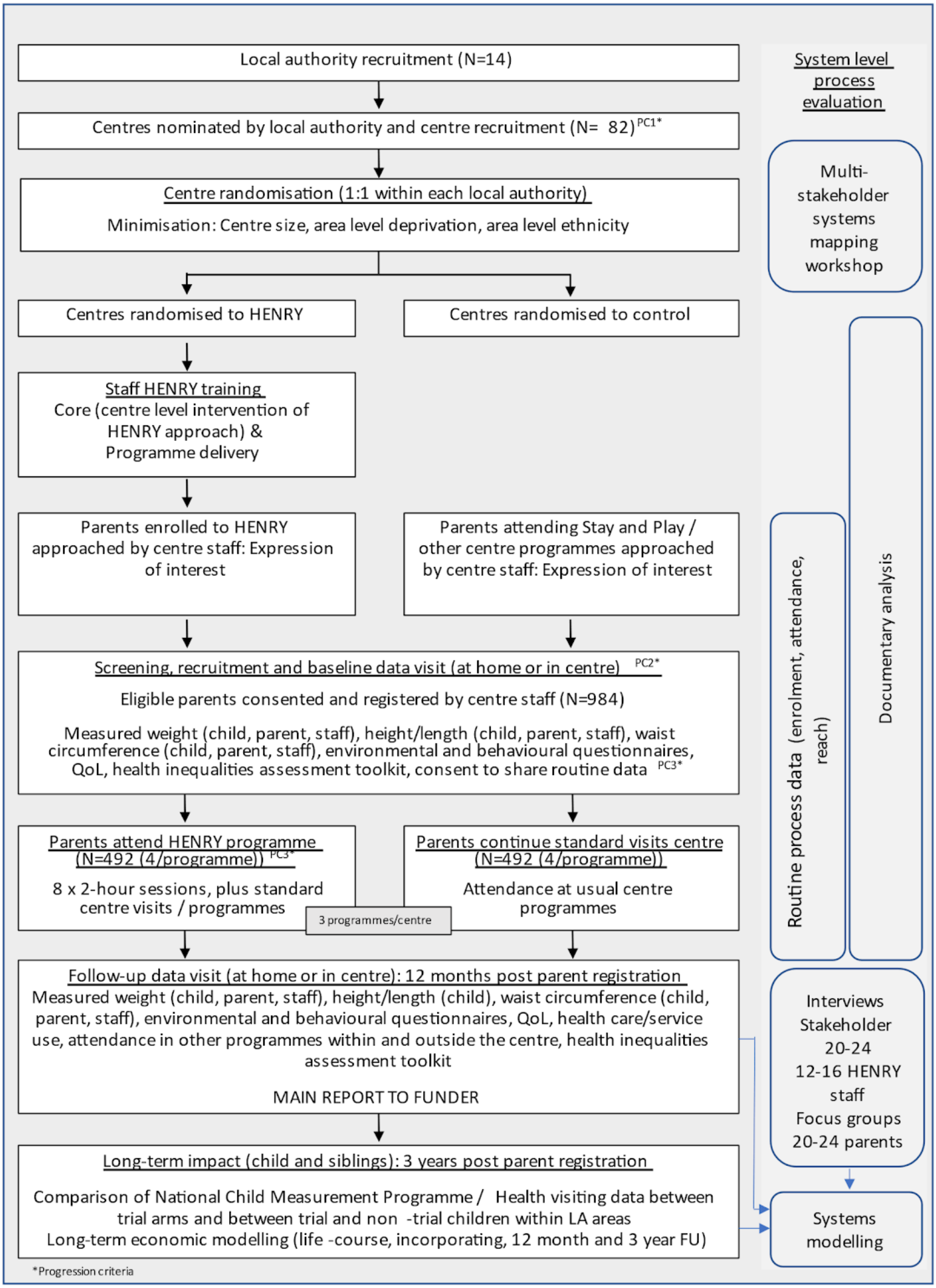

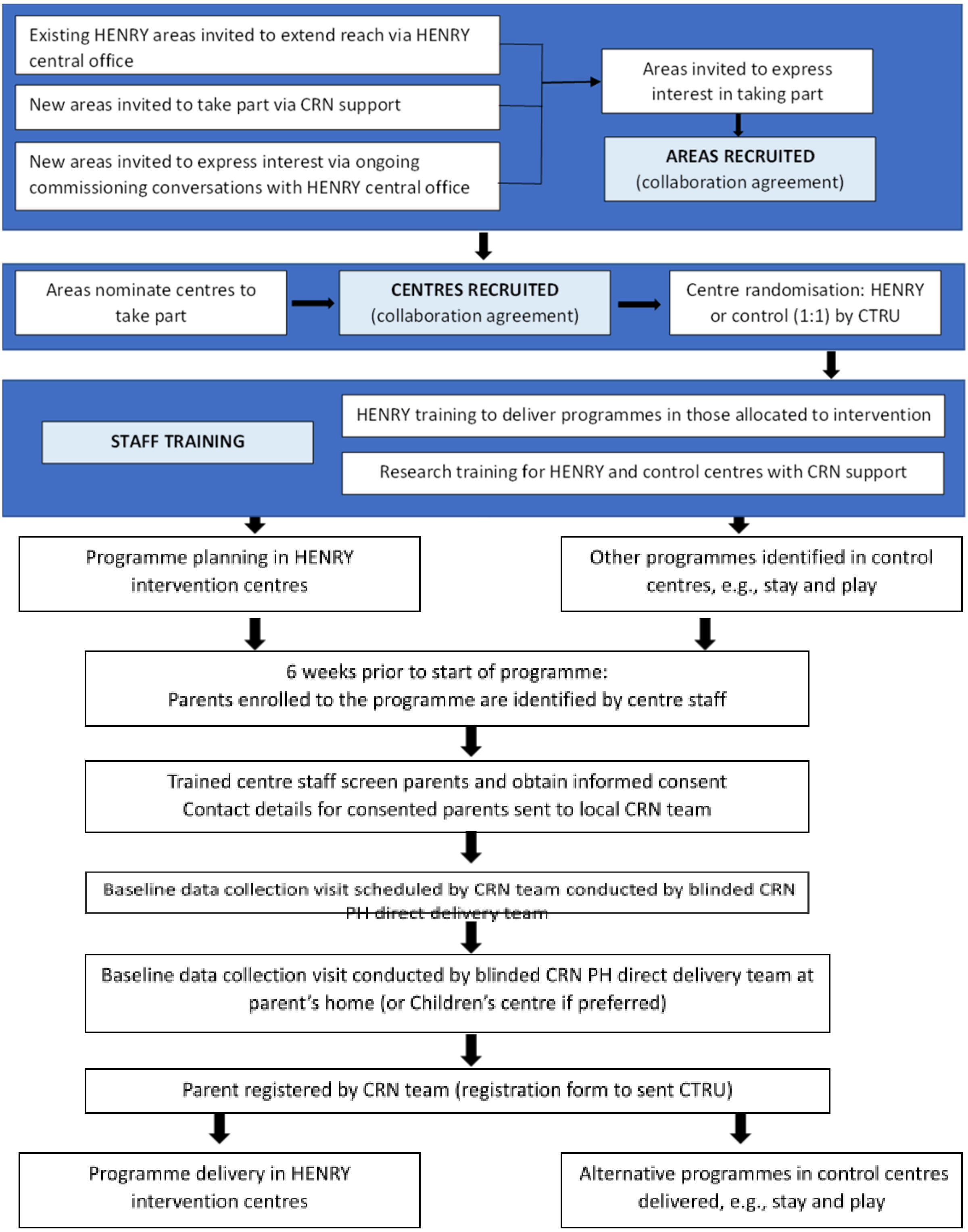
Existing HENRY areas invited to extend reach via HENRY central office
New areas invited to take part via CRN support
New areas invited to express interest via ongoing commissioning conversations with HENRY central office
Areas invited to express interest in taking part
AREAS RECRUITED (collaboration agreement)
Areas nominate centres to take part
CENTRES RECRUITED (collaboration agreement)
Centre randomisation: HENRY or control (1:1) by CTRU
STAFF TRAINING
HENRY training to deliver programmes in those allocated to intervention
Research training for HENRY and control centres with CRN support
Programme planning in HENRY intervention centres
Other programmes identified in control centres, e.g., stay and play
6 weeks prior to start of programme: Parents enrolled to the programme are identified by centre staff
Trained centre staff screen parents and obtain informed consent
Contact details for consented parents sent to local CRN team
Baseline data collection visit scheduled by CRN team conducted by blinded CRN PH direct delivery team
Baseline data collection visit conducted by blinded CRN PH direct delivery team at parent's home (or Children's centre if preferred)
Parent registered by CRN team (registration form to sent CTRU)
Programme delivery in HENRY intervention centres
Alternative programmes in control centres delivered, e.g., stay and play

Supplementary Table 1 Data collection summary

**Data/outcomes**	**Measures**	Collected by	LA[c] / CC[d] Screening and consent	Centre baseline	Participant screening	Participant informed consent and registration	Participant / staff Baseline	Short term follow-up (12 months post registration)	Medium-term follow-up (3 years)[a]	Longer term follow-up[b]
**Local authority**										
Eligibility	LA Eligibility checklist	LA self complete	x							
Basic demographics / reasons for declining	LA / CC Recruitment log	Researcher / HENRY	x							
Centre contact details	Centre nomination form	LA self complete	x							
Local authority agreement / consent	Local authority agreement	LA self complete	x							
**Centre**										
Eligibility	LA Eligibility checklist	LA self complete	x							
Reasons for declining / ineligibility	LA / CC recruitment log	Researcher	x							
Centre agreement / consent	Children's Centre agreement	Centre self complete	x							

For peer review only

**Data/outcomes**	**Measures**	Collected by	LA[c] / CC[d] Screening and consent	Centre baseline	Participant screening	Participant informed consent and registration	Participant / staff Baseline	Short term follow-up (12 months post registration)	Medium-term follow-up (3 years)[a]	Longer term follow-up[b]
Stratification factors	Children's centre nomination form	LA self complete		x						
Centre randomisation allocation	Randomisation form	CTRU[e]		x						
Demographics	Centre nomination form / Environmental Questionnaire	LA / Centre self complete		x						
Details of other courses	Environmental Questionnaire	Centre self complete		x				x	x	
**Parent**										
Initial eligibility check	Screening form	Centre staff			x					
Confirmation of eligibility	Eligibility checklist	LCRN[f]				x				
Reasons for declining / ineligibility	Screening form	Parent self complete			x					
Demographics (parent, child and family)	Screening form Baseline form	Parent self complete CTRU (recruitment log)			x		x			

For peer review only

**Data/outcomes**	**Measures**	Collected by	LA[c] / CC[d] Screening and consent	Centre baseline	Participant screening	Participant informed consent and registration	Participant / staff Baseline	Short term follow-up (12 months post registration)	Medium-term follow-up (3 years)[a]	Longer term follow-up[b]
Consent to trial participation and data collection (including NCMP)	Consent form	Parent self complete				x				
Use of Centres	Baseline form Follow up form	LCRN					x	x	x	
**Outcomes**										
Child age and sex adjusted BMI Z-score, child height, child weight, unadjusted BMI and weight/BMI percentiles	Measured	LCRN (baseline & 12 M follow-up)<br><br>Longer term: CTRU accessing routine data					x	x	x	
Sibling age and sex adjusted BMI, height (m), weight (kg), unadjusted BMI and weight/BMI percentiles		CTRU accessing routine data							x	
Parent self-efficacy	Dumka (27)	LCRN					x	x		
Family eating / activities	Golan (28)	LCRN					x	x		
Feeding questionnaire	Baughcum (29)	LCRN					x	x		

For peer review only

**Data/outcomes**	**Measures**	Collected by	LA[c] / CC[d] Screening and consent	Centre baseline	Participant screening	Participant informed consent and registration	Participant / staff Baseline	Short term follow-up (12 months post registration)	Medium-term follow-up (3 years)[a]	Longer term follow-up[b]
Dental health (child)	Dental questionnaire	LCRN					x	x		
Parent height & weight:	Measured	LCRN					x	x		
Parent waist circumference	Measured	LCRN					x	x		
**Staff:**										
Staff screening	Staff screening form	Staff self complete at centre		x						
Staff height & weight	Measured	Self-measure					x	x		
Staff waist circumference	Measured	Self-measure					X	x		
**Children's Centre:**										
Centre social, physical and political environment (e.g. policies around food)	Environmental questionnaire	Centre self complete		x				x		
**Safety:**										

For peer review only

**Data/outcomes**	**Measures**	Collected by	LA[c] / CC[d] Screening and consent	Centre baseline	Participant screening	Participant informed consent and registration	Participant / staff Baseline	Short term follow-up (12 months post registration)	Medium-term follow-up (3 years)[a]	Longer term follow-up[b]
Adverse events / unintended consequences	SAE / RUSAE forms	LCRN / centre self complete						x		
**Health economics:**										
Health care resource use for child	Resource use questionnaire	LCRN					x	x		
Health care resource use for parents	Resource use questionnaire	LCRN					x	x		
Private costs (e.g. travel costs, additional food expenses, lost productivity)	Resource use questionnaire	LCRN					x	x		
Parent's Health-related Quality of Life	EQ-5D-5L (30) and ICECAP-A [31]	LCRN					x	x		
**Routine data:**										
NCMP Child and sibling data (trial participants)		CTRU							x	
NCMP regional child data (not trial participants)		CTRU							x	

For peer review only

**Data/outcomes**	**Measures**	Collected by	LA[c] / CC[d] Screening and consent	Centre baseline	Participant screening	Participant informed consent and registration	Participant / staff Baseline	Short term follow-up (12 months post registration)	Medium-term follow-up (3 years)[a]	Longer term follow-up[b]
Process evaluation/delivery of HENRY programmes:										
Attendance * *Data collection not timed to trial time points	HENRY attendance records	HENRY Centre staff / HENRY facilitator								
Contamination checks* *Data collection not timed to trial time points	Staff movement form	Staff self complete								
HENRY training attendance and knowledge		HENRY						x	x	
**Qualitative data**										
System map and sub maps* *Data collection not timed to trial time points	n/a	Researcher								
Systems mapping interviews with local authority stakeholders* *Data collection not timed to trial time points	n/a	Researcher								

For peer review only

**Data/outcomes**	**Measures**	Collected by	LA[c] / CC[d] Screening and consent	Centre baseline	Participant screening	Participant informed consent and registration	Participant / staff Baseline	Short term follow-up (12 months post registration)	Medium-term follow-up (3 years)[a]	Longer term follow-up[b]
Interviews with centre staff* *Data collection not timed to trial time points	n/a	Researcher								
Interviews with parents* **Data collection not timed to trial time points	n/a	Researcher								

[a] Medium term outcomes will gathered from routinely collected data (from health visitors and/or the National Child Measurement Programme)

[b] Longer term outcomes will be based on matched cohorts of (Millennium Cohort Study (MCS ((21)).

[C] Local Authority (or other governing/commissioning body)

[d] Children's centre (or other setting in which HENRY can be delivered, including Sure Start centres in Northern Ireland)

[e] Clinical Trials Research Unit

[f] Local Clinical Research Network (England only). Other devolved nations will train researchers/equivalent staff to collect data)

For peer review only

For peer review only

STANDARD PROTOCOL ITEMS: RECOMMENDATIONS FOR INTERVENTIONAL TRIALS

SPIRIT 2013 Checklist: Recommended items to address in a clinical trial protocol and related documents*
NB: Items with a 'RP' response are available on the registered protocol (ISRCTN16529380)

Section/item	Item No	Description	Addressed on page number
**Administrative information**			
Title	1	Descriptive title identifying the study design, population, interventions, and, if applicable, trial acronym	___1__________
Trial registration	2a	Trial identifier and registry name. If not yet registered, name of intended registry	___2__________
	2b	All items from the World Health Organization Trial Registration Data Set	_____________
Protocol version	3	Date and version identifier	__RP________
Funding	4	Sources and types of financial, material, and other support	___6__________
Roles and responsibilities	5a	Names, affiliations, and roles of protocol contributors	___1,6________
	5b	Name and contact information for the trial sponsor	___RP_________
	5c	Role of study sponsor and funders, if any, in study design; collection, management, analysis, and interpretation of data; writing of the report; and the decision to submit the report for publication, including whether they will have ultimate authority over any of these activities	___RP_______

For peer review only - http://bmjopen.bmj.com/site/about/guidelines.xhtml

	5d	Composition, roles, and responsibilities of the coordinating centre, steering committee, endpoint adjudication committee, data management team, and other individuals or groups overseeing the trial, if applicable (see Item 21a for data monitoring committee)	___RP______
**Introduction**			
Background and rationale	6a	Description of research question and justification for undertaking the trial, including summary of relevant studies (published and unpublished) examining benefits and harms for each intervention	___4,5________
	6b	Explanation for choice of comparators	____9_________
Objectives	7	Specific objectives or hypotheses	____4,5________
Trial design	8	Description of trial design including type of trial (eg, parallel group, crossover, factorial, single group), allocation ratio, and framework (eg, superiority, equivalence, noninferiority, exploratory)	____5_________
**Methods: Participants, interventions, and outcomes**			
Study setting	9	Description of study settings (eg, community clinic, academic hospital) and list of countries where data will be collected. Reference to where list of study sites can be obtained	____5,6________
Eligibility criteria	10	Inclusion and exclusion criteria for participants. If applicable, eligibility criteria for study centres and individuals who will perform the interventions (eg, surgeons, psychotherapists)	____6_________
Interventions	11a	Interventions for each group with sufficient detail to allow replication, including how and when they will be administered	____8,9________
	11b	Criteria for discontinuing or modifying allocated interventions for a given trial participant (eg, drug dose change in response to harms, participant request, or improving/worsening disease)	____NA________
	11c	Strategies to improve adherence to intervention protocols, and any procedures for monitoring adherence (eg, drug tablet return, laboratory tests)	____20________
	11d	Relevant concomitant care and interventions that are permitted or prohibited during the trial	____NA______

For peer review only - http://bmjopen.bmj.com/site/about/guidelines.xhtml

Outcomes	12	Primary, secondary, and other outcomes, including the specific measurement variable (eg, systolic blood pressure), analysis metric (eg, change from baseline, final value, time to event), method of aggregation (eg, median, proportion), and time point for each outcome. Explanation of the clinical relevance of chosen efficacy and harm outcomes is strongly recommended	___9-16______
Participant timeline	13	Time schedule of enrolment, interventions (including any run-ins and washouts), assessments, and visits for participants. A schematic diagram is highly recommended (see Figure)	___Figure 1_____
Sample size	14	Estimated number of participants needed to achieve study objectives and how it was determined, including clinical and statistical assumptions supporting any sample size calculations	___17____
Recruitment	15	Strategies for achieving adequate participant enrolment to reach target sample size	___6-7________

Methods: Assignment of interventions (for controlled trials)

Allocation:

Sequence generation	16a	Method of generating the allocation sequence (eg, computer-generated random numbers), and list of any factors for stratification. To reduce predictability of a random sequence, details of any planned restriction (eg, blocking) should be provided in a separate document that is unavailable to those who enrol participants or assign interventions	___7________
Allocation concealment mechanism	16b	Mechanism of implementing the allocation sequence (eg, central telephone; sequentially numbered, opaque, sealed envelopes), describing any steps to conceal the sequence until interventions are assigned	____RP________
Implementation	16c	Who will generate the allocation sequence, who will enrol participants, and who will assign participants to interventions	____RP________
Blinding (masking)	17a	Who will be blinded after assignment to interventions (eg, trial participants, care providers, outcome assessors, data analysts), and how	____7-8________
	17b	If blinded, circumstances under which unblinding is permissible, and procedure for revealing a participant's allocated intervention during the trial	____RP________

Methods: Data collection, management, and analysis

Data collection methods	18a	Plans for assessment and collection of outcome, baseline, and other trial data, including any related processes to promote data quality (eg, duplicate measurements, training of assessors) and a description of study instruments (eg, questionnaires, laboratory tests) along with their reliability and validity, if known. Reference to where data collection forms can be found, if not in the protocol	___17 & Table 1_
	18b	Plans to promote participant retention and complete follow-up, including list of any outcome data to be collected for participants who discontinue or deviate from intervention protocols	____17_______
Data management	19	Plans for data entry, coding, security, and storage, including any related processes to promote data quality (eg, double data entry; range checks for data values). Reference to where details of data management procedures can be found, if not in the protocol	__RP____
Statistical methods	20a	Statistical methods for analysing primary and secondary outcomes. Reference to where other details of the statistical analysis plan can be found, if not in the protocol	___17-18_____
	20b	Methods for any additional analyses (eg, subgroup and adjusted analyses)	__18__
	20c	Definition of analysis population relating to protocol non-adherence (eg, as randomised analysis), and any statistical methods to handle missing data (eg, multiple imputation)	__17________

Methods: Monitoring

Data monitoring	21a	Composition of data monitoring committee (DMC); summary of its role and reporting structure; statement of whether it is independent from the sponsor and competing interests; and reference to where further details about its charter can be found, if not in the protocol. Alternatively, an explanation of why a DMC is not needed	__21-22______
	21b	Description of any interim analyses and stopping guidelines, including who will have access to these interim results and make the final decision to terminate the trial	___17________
Harms	22	Plans for collecting, assessing, reporting, and managing solicited and spontaneously reported adverse events and other unintended effects of trial interventions or trial conduct	__21 & Table 1_
Auditing	23	Frequency and procedures for auditing trial conduct, if any, and whether the process will be independent from investigators and the sponsor	___21________

Ethics and dissemination

For peer review only - http://bmjopen.bmj.com/site/about/guidelines.xhtml

Research ethics approval	24	Plans for seeking research ethics committee/institutional review board (REC/IRB) approval	___2________
Protocol amendments	25	Plans for communicating important protocol modifications (eg, changes to eligibility criteria, outcomes, analyses) to relevant parties (eg, investigators, REC/IRBs, trial participants, trial registries, journals, regulators)	__RP_____
Consent or assent	26a	Who will obtain informed consent or assent from potential trial participants or authorised surrogates, and how (see Item 32)	__6 & Table 1_
	26b	Additional consent provisions for collection and use of participant data and biological specimens in ancillary studies, if applicable	____NA______
Confidentiality	27	How personal information about potential and enrolled participants will be collected, shared, and maintained in order to protect confidentiality before, during, and after the trial	_____RP_____
Declaration of interests	28	Financial and other competing interests for principal investigators for the overall trial and each study site	_____22_____
Access to data	29	Statement of who will have access to the final trial dataset, and disclosure of contractual agreements that limit such access for investigators	____RP_______
Ancillary and post-trial care	30	Provisions, if any, for ancillary and post-trial care, and for compensation to those who suffer harm from trial participation	____NA________
Dissemination policy	31a	Plans for investigators and sponsor to communicate trial results to participants, healthcare professionals, the public, and other relevant groups (eg, via publication, reporting in results databases, or other data sharing arrangements), including any publication restrictions	____21_______
	31b	Authorship eligibility guidelines and any intended use of professional writers	___RP________
	31c	Plans, if any, for granting public access to the full protocol, participant-level dataset, and statistical code	___NA________
**Appendices**			
Informed consent materials	32	Model consent form and other related documentation given to participants and authorised surrogates	___RP________

For peer review only - http://bmjopen.bmj.com/site/about/guidelines.xhtml

Biological specimens	33	Plans for collection, laboratory evaluation, and storage of biological specimens for genetic or molecular analysis in the current trial and for future use in ancillary studies, if applicable	____NA_______

*It is strongly recommended that this checklist be read in conjunction with the SPIRIT 2013 Explanation & Elaboration for important clarification on the items. Amendments to the protocol should be tracked and dated. The SPIRIT checklist is copyrighted by the SPIRIT Group under the Creative Commons "Attribution-NonCommercial-NoDerivs 3.0 Unported" license.

For peer review only
